# Development of an Advanced Manufacturing Technology for Continuous Drug Substance Production

**DOI:** 10.1021/acs.iecr.5c04185

**Published:** 2026-01-13

**Authors:** Daniel G. Gregory, Kaitlin E. Kay, Justin T. Turnage, Kimberly E. Penzer, James K. Ferri

**Affiliations:** Department of Chemical and Life Science Engineering, Virginia Commonwealth University, 601 West Main St., Richmond, Virginia 23284, United States

## Abstract

We describe the early conceptual development of a candidate advanced manufacturing technology (AMT) which enables the synthesis of albuterol sulfate, a bronchodilator used for the treatment of asthma, and an API currently listed on the FDA’s drug shortage list. The candidate AMT system is currently under construction for the automated production of +2,000 mg/h of albuterol sulfate via a new synthetic pathway with a 78.4% solution yield when operating at a 1.0 mL/min flow rate basis. Additionally, system throughput can be scaled 10-fold with minor modifications. The authors plan to apply for AMT approval of this technology under the FDA’s new AMT designation program. Key engineering design strategies are highlighted for successful translation of traditional batch synthetic methods toward continuous manufacturing, with an emphasis placed on process intensification via rational synthon selection, the introduction of continuous flow technologies, incorporation of in-line process analytical technologies (PAT), and system scale-up within a larger production facility. Analytical characterization via high-performance liquid chromatography (HPLC), LC mass spectrometry (LC-MS), gas chromatography–flame ionization detection (GC-FID), and in-line nuclear magnetic resonance spectroscopy (NMR) are utilized to assess composition and purity throughout the process. The results presented herein enable scale-up of an automated continuous manufacturing system as it provides a means of exceeding batch efficiency during the production of liquid drug formulations; a strategy which can reduce capital costs, eliminate drug shortages, and strengthen America’s pharmaceutical supply chain resiliency.

## Graphical abstract

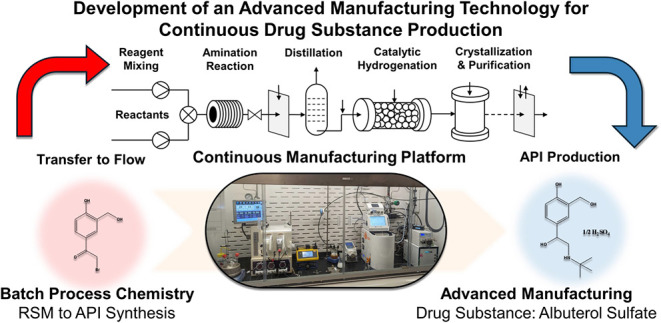

## Introduction

Pharmaceutical manufacturers have continually developed remarkable drug innovations which enable the life-saving treatment of an ever-growing range of diseases. However, the strategic U.S. pharmaceutical manufacturing landscape has gradually eroded in recent decades and has culminated in the widespread shortage of critical life-saving generic drugs.^1^ As pharmaceutical patents age and eventually expire, the production of off-patent generic drugs becomes increasingly unprofitable.^2^ These economic realities have driven U.S.-based pharmaceutical manufacturers to focus increased attention and resources on new drug development for novel blockbuster therapeutics, rather than facilitating the production of generic life-saving drugs with marginal profitability.^3^

The narrow profit margins associated with generic drugs have led to fierce competition between manufacturers, a phenomenon which has led to the rapid off shoring of U.S.-based drug manufacturing. Today, over +80% of active pharmaceutical ingredients (API’s) are manufactured overseas,^4^ a reality which has hindered the U.S. Food and Drug Administration’s (FDA) ability to verify the purity of regulatory starting materials (RSM’s) and API’s.^5^ This has resulted in supply chain delays and chronic drug shortages for critical life-saving drugs such as albuterol sulfate.^6^ Fortunately, a new FDA designation seeks to employ advanced manufacturing technologies (AMT’s) to continuously manufacture key life-sustaining drugs via new methods of continuous flow automation.^7^

Active pharmaceutical ingredients are conventionally synthesized via batch processes on an industrial scale which enable the production of up to 1,000 t of API per year.^8–10^ Although batch processing endures as the industry standard, its associated costs and inherent necessity for hands-on labor have driven generic API manufacturing offshore while extending complex logistical networks which span across multiple continents.^1,4,11,12^ These manufacturing trends are now a global phenomenon that require detailed chain-of-custody documentation which complicates the ability of regulatory bodies both domestically and abroad (e.g., the European Medicines Agency) to monitor drug purity. This challenge encompasses the entire life cycle of the manufacturing process, ranging from RSM to API synthesis, and ultimately packaging of the final drug product.^13,14^ Consequently, the inefficiencies associated with batch manufacturing have led to global supply chain delays for critical life-saving drugs, a phenomenon which was recently exacerbated by the SARS-CoV-2 pandemic.^15^

A new and emerging field seeks to employ advanced manufacturing technologies for the production of vital life-saving drugs via continuous flow synthesis and drug product manufacturing.^8,16,17^ AMT’s are defined as a method of manufacturing which incorporates a novel technology that will substantially improve the pharmaceutical manufacturing process by (I) reducing development time for a drug or (II) increasing or maintaining the supply of a drug listed on the drug shortage list, as defined by section §506E of the FD&C Act (21 U.S.C. §356e).^7,18^ This national strategy has the potential to automate drug manufacturing, bolster strategic pharmaceutical stockpiles, and eliminate drug shortages.^19,20^

Advanced pharmaceutical manufacturing systems can be configured for continuous high-throughput production of drug products and can be monitored with in-line process analytical technologies (PAT) to ensure drug substance purity immediately prior to packaging.^21–24^ However, further research and development is necessary to design, engineer, and implement this emerging technology. This manuscript highlights the ongoing construction of a candidate AMT system for the advanced manufacturing of albuterol sulfate (CAS# 51022–70–9), a bronchodilator asthma drug on the FDA’s drug shortage list.^25–27^ Here we detail our initial laboratory research, laboratory scale prototype systems, and conceptual development of a candidate AMT system which is currently under construction for the automated production of albuterol sulfate. The automated pilot scale AMT system is currently under construction at a contract manufacturing organization facility (CMO), and the team will submit both an AMT application and abbreviated new drug approval (ANDA) for the manufacture of albuterol sulfate upon its completion.

Albuterol sulfate, i.e., Salbutamol, is a short-acting β-adrenergic (SABA) receptor agonist commonly used to relieve bronchospasms during the treatment of chronic lung conditions including asthma, emphysema, and chronic obstructive pulmonary disease (i.e., COPD).^28,29^ The drug was first disclosed in a series of Nature articles in 1968 and was subsequently patented by Allen and Hanburys.^30–34^ Albuterol was initially marketed as Ventolin during the height of the 1960s U.K. asthma epidemic and its widespread prescription led to a rapid reduction in asthma-related deaths throughout the 1970s.^35^ This innovative asthma therapy was administered without the negative cardiac side effects common to alternative asthma drugs of the era including adrenaline and isoprenaline.^36^

Throughout the 1970s and 80s albuterol-related research focused primarily on new routes of synthesis, chiral pharmacophore activity, the incorporation of long-acting β-agonists (LABA's), and new methods of pulmonary drug delivery.^37^ Investigations into the therapeutically active conformation of the drug (i.e., R- versus S-) have been inconclusive and a debate remains regarding which conformation is most active.^38–42^ Thus, this life-saving drug is typically packaged and sold as a racemic mixture which is delivered to the patient via an inhaler or in a liquid dosage form.^43–47^ A standard albuterol sulfate dose typically ranges from 0.2–6.0 mg per dose depending on the size and age of the patient.^48^

Albuterol sulfate has now been successfully utilized for the treatment of asthma and COPD for over +50 years. Yet despite this remarkable track record of success, the economically advantageous synthetic pathways toward batch albuterol manufacturing have been largely exhausted in recent decades.^49^ Meanwhile the gradual expiration of albuterol-related patents has reduced the overall profitability of its production, a problematic trend common to off-patent API’s and generic drugs.^50^ This profitability-related phenomenon has driven the industrial production of API’s, including albuterol sulfate, to overseas manufacturers.^2^ This dynamic often corresponds with abrupt supply chain disruptions as was recently emphasized by the bankruptcy of Akorn Pharmaceuticals, a large U.S.-based albuterol manufacturer; thus, the U.S. is currently left with only one remaining domestic albuterol manufacturer (i.e., Nephron Pharmaceuticals).^3^

Continuous AMT’s can reduce manufacturing costs by incorporating process intensification (PI), reducing waste, and enabling new synthetic pathways.^51^ While the traditional batch pathways for albuterol manufacturing offer marginal profitability, advanced manufacturing via automated continuous flow synthesis offers a compelling new avenue toward API production. Here we utilize a new salicylaldehyde-based route to complete the synthesis of albuterol sulfate within continuous-flow prototype reactor systems. A comprehensive literature search reveals that albuterol has primarily been commercially produced in batch processes from regulatory starting materials (RSM) including p-hydroxyacetophenones and salicylic acid derivatives including salicylaldehyde (CAS# 90-02-8).^52–57^ In recent decades, the synthetic route starting from salicylaldehyde has become the primary commercial route of manufacturing.^56^ These batch synthetic processes often involve the incorporation of large protecting groups, repetitive isolation steps, and overall isolated yields ranging from ∼20–43%.^58^

The continuous API manufacturing system detailed herein incorporates a series of commercial flow chemistry platforms, in-line PAT’s, and in-house solutions which facilitate a telescoped three-step process which enables the production of +2,000 mg of albuterol sulfate per hour and a 78.4% API solution yield. The overall candidate AMT process can generate +700 doses per hour at a 1.0 mL/min basis for use in a liquid dose albuterol sulfate drug formulation (i.e., 3.0 mg in a 3.0 mL aqueous solution). Each modular unit operation in this text has been individually developed and optimized for configuration in a prototype AMT system. Key chemistry, unit operation, and engineering decisions are highlighted throughout the text including the incorporation of a tubular flow reactor to enable S_N_2 amination, a catalytic packed bed reactor (PBR) for hydrogenation, continuous sulfation, and a series of purification submodules consisting of dead-end filters, in-line distillation, and a separate off-line Nutsche-style filter dryer.

Analytical drug product purity was orthogonally validated by correlating off-line analytical characterization via high performance liquid chromatography (HPLC), LC mass spectrometry (LC-MS), gas chromatography–flame ionization detection (GC-FID), and proton nuclear magnetic resonance spectroscopy (^1^H NMR). A discussion involving the future integration of ^1^H NMR as an in-line PAT is included. Lastly, a forward-looking assessment involving the incorporation of an automated laboratory filter dryer (LFD) is examined to enable 10-fold scale-up and seamless transition between synthesis and purification submodules. Finally, the system will be integrated end-to-end with a second modular system for encapsulation of the finished drug product. This collaborative ongoing work is being conducted with colleagues at the Center for Structured Organic Particulate Systems (i.e., C-SOPS), who have realized pioneering solutions for continuous drug manufacturing.^17,59–64^ Ultimately, the advanced manufacturing of strategic API’s using the engineered AMT solutions detailed within this article will help reduce drug costs and will bolster America’s pharmaceutical supply chain resiliency.

## Process Overview

The overarching vision of this emerging AMT design was to develop a standalone modular system capable of the synthesis of liquid-dose pharmaceutical products (i.e., the asthma drug albuterol sulfate) via a continuous API manufacturing system. An initial series of batch chemistry studies were performed to develop a new and innovative synthetic route which could be translated into a continuous flow process. Next, these chemical transformations were screened within modular continuous flow unit operations to optimize the process conditions necessary for continuous manufacturing (see Discussion). Finally, the unit operations were assembled into modular prototype systems and scaled to meet target throughput requirements within a pilot plant. The conceptual AMT system detailed within this text can be assembled from commercial flow chemistry reactors and in-house solutions to achieve a multistep telescoped process for the synthesis of albuterol sulfate (see Figure 1).

**Figure 1:**
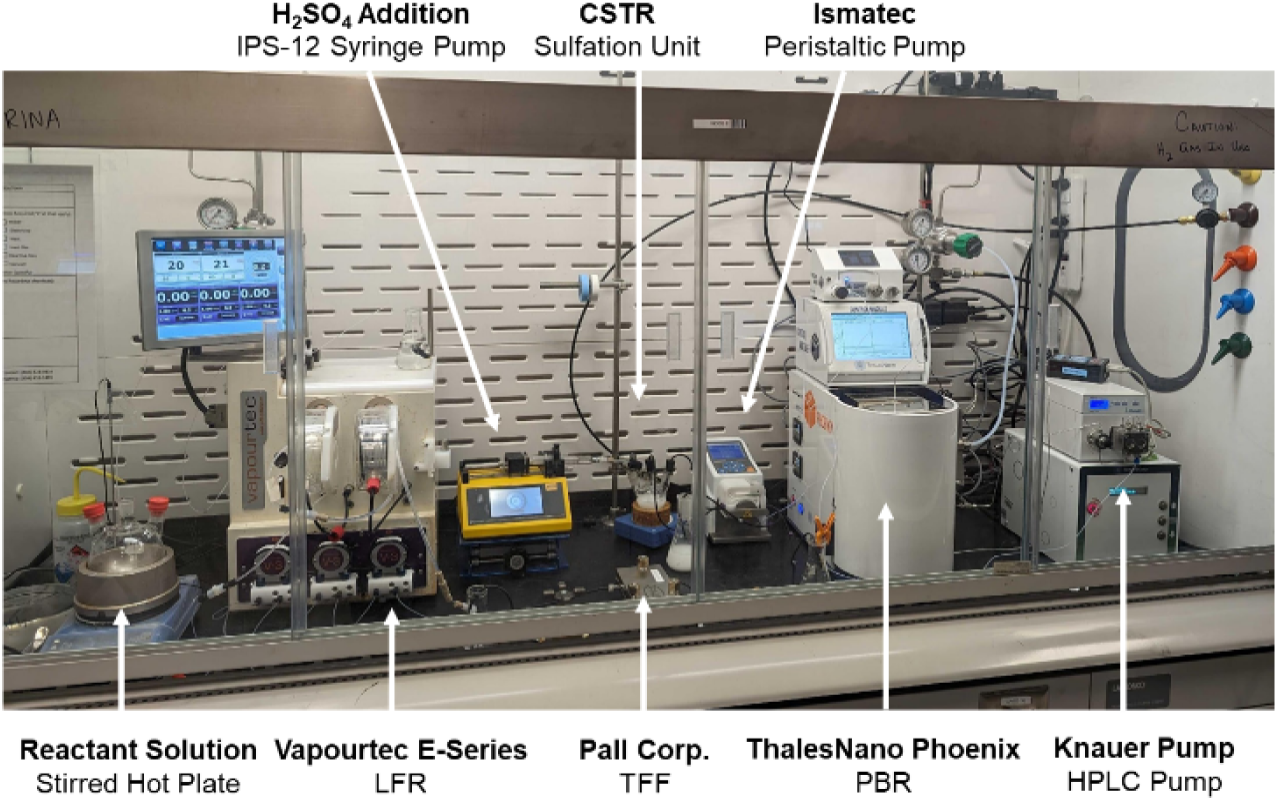
A modular flow system for the continuous production of albuterol sulfate in flow. The system depicted here includes reactant vessels, a Vaportec E-Series flow reactor (Step 1: Amination), a tangential flow filter (TFF), a ThalesNano Phoenix catalytic reactor (Step 2: Hydrogenation), and a continuous CSTR sulfation system (Step 3: API Salt Formation).

The synthetic procedure developed for the AMT process involves a telescoped three-step transformation of a bromo-diol-based molecular starting material (SM) i.e., 2-Bromo-1­[4-hydroxy-3-(hydroxymethyl)­phenyl]­ethan-1-one into albuterol sulfate (see Figure 2). This synthetic pathway is first accomplished through an S_N_2 amination reaction to form a key chemical intermediate (i.e., Figure 2, Molecule 2). Next, a heterogeneous catalytic hydrogenation reaction is utilized to convert the intermediate into albuterol freebase as shown in Figure 2, Molecule (3). The final transformation involves conversion of the freebase into the API salt, albuterol sulfate. The API is then readily purified within common semibatch purification methods involving filtration and recrystallization.

**Figure 2:**
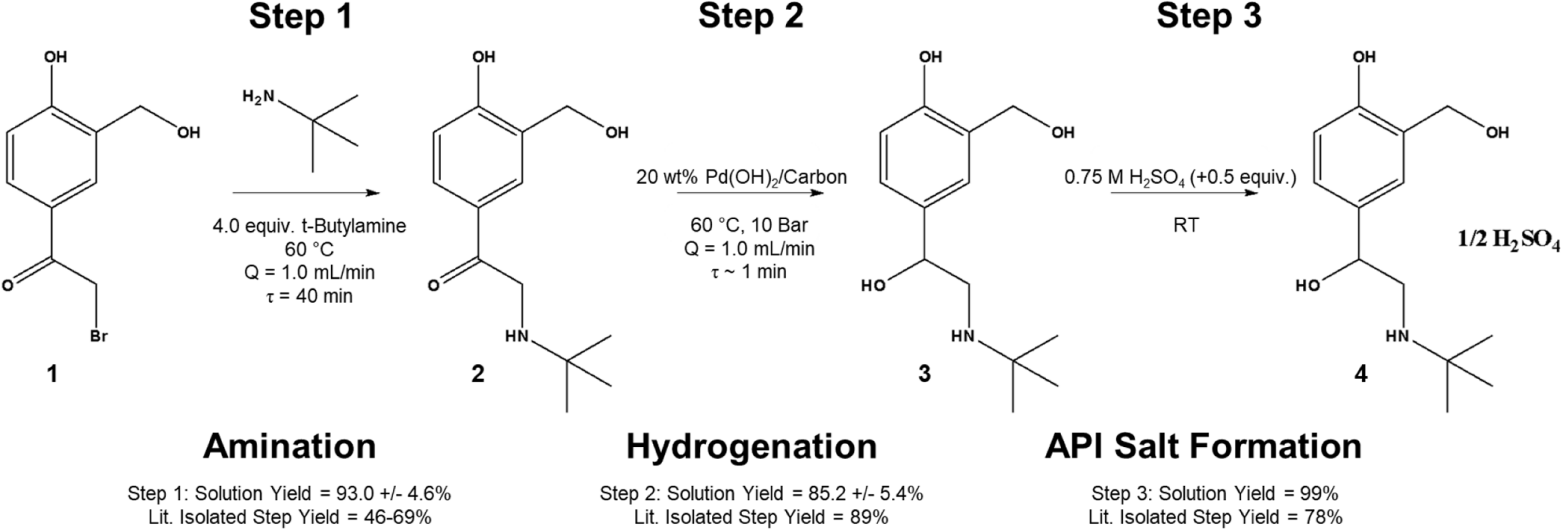
New process chemistry route utilized for the production of albuterol sulfate.

This new synthetic pathway was developed for operation within a prototype laboratory AMT system as described within this text and is currently under development for integration within a scaled-up, automated pilot plant. A simplified process flow diagram (PFD) for the fully automated, continuous albuterol sulfate manufacturing system is presented in Figure 3. The key unit operations include an S_N_2 amination reaction within a laminar flow reactor (LFR); in-line distillation, hydrogenation through a packed bed reactor (PBR); and sulfation within a continuously stirred tank reactor (CSTR). The final active pharmaceutical ingredient can then be purified via vacuum filtration and recrystallization within a Nutsche-style filter dryer. These continuous processing steps are briefly described in the following sections.

**Figure 3:**
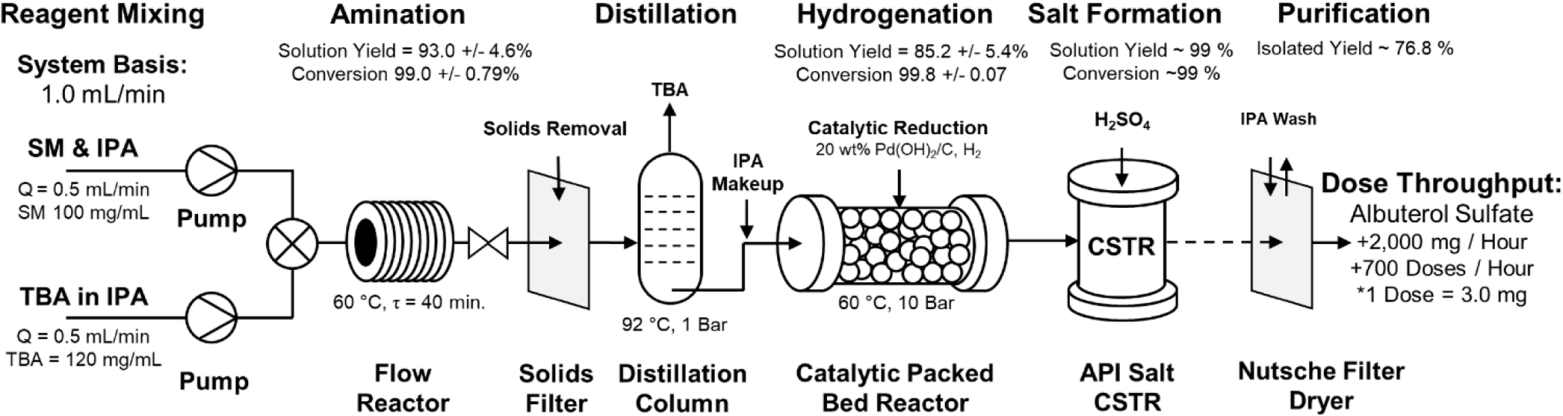
A simplified process flow diagram for albuterol sulfate manufacturing within a candidate AMT pilot plant (Basis = 1.0 mL/min).

## Process Optimization of Unit Operations

### Amination of Starting Material

In the first step of the process, the bromo-diol SM **(1)**, was utilized to synthesize a key chemical intermediate **(2)** within an LFR. The bromo-diol SM **(1)** consisted of a light brown solid which readily mixed in common alcohol solvents including methanol (MeOH) and isopropanol (IPA). The SM **(1)** was transformed through an S_N_2 amination reaction with four equivalents of *tert*-butylamine to form the intermediate albuterol species **(2)**. Complementary analytical characterization was completed with liquid chromatography mass spectrometry (LC-MS) and proton nuclear magnetic resonance spectroscopy. The primary impurities generated by the S_N_2 amination reaction included a series of t-butylamine salts and polymeric intermediates consisting of dimerized and trimerized species (see SI Figures S1–S4).^65^

After batch validation of the new synthetic pathway, the continuous amination reaction was conducted within a commercial Vaportec E-Series laminar flow reactor equipped with 1/16 in. ID tubing. To charge the reactor, both the bromo-diol starting material **(1)** and t-butylamine were mixed in extra dry process solvent (i.e., MeOH or IPA). The reactants were stored in capped round-bottom flasks (RBF) under inert nitrogen as the amination reaction is susceptible to degradation when in the presence of air and moisture. The solutions were then pumped from their respective flasks at a total flow rate (Q) of 1.0 mL per min (i.e., 0.5 mL/min each) and subsequently mixed along a T-shaped mixing junction which was located just prior to the entry of the LFR.

The amination reaction was initially screened at temperatures ranging from 25 to 60 °C and flow rates ranging from 0.3 to 10 mL/min (see Figure 4). During the initial screening study, continuous process parameters consisting of a 40 min residence time (τ), a 60 °C reactor temperature, and a flow rate of 1.0 mL/min in IPA achieved a 93.0 ± 4.6% solution yield of molecule **(2)** and 99.0 ± 0.79% conversion of the SM **(1)** at a 95% confidence interval (CI) as assessed with HPLC. Conversely, at low residence times (τ < 30 min) and low temperatures (*T* < 60 °C) incomplete conversions and solution yields were obtained. Additionally, the utilization of IPA as a process solvent demonstrated both increased yield and conversion in comparison to methanol. See Penzer et al. for further information.^66^

**Figure 4:**
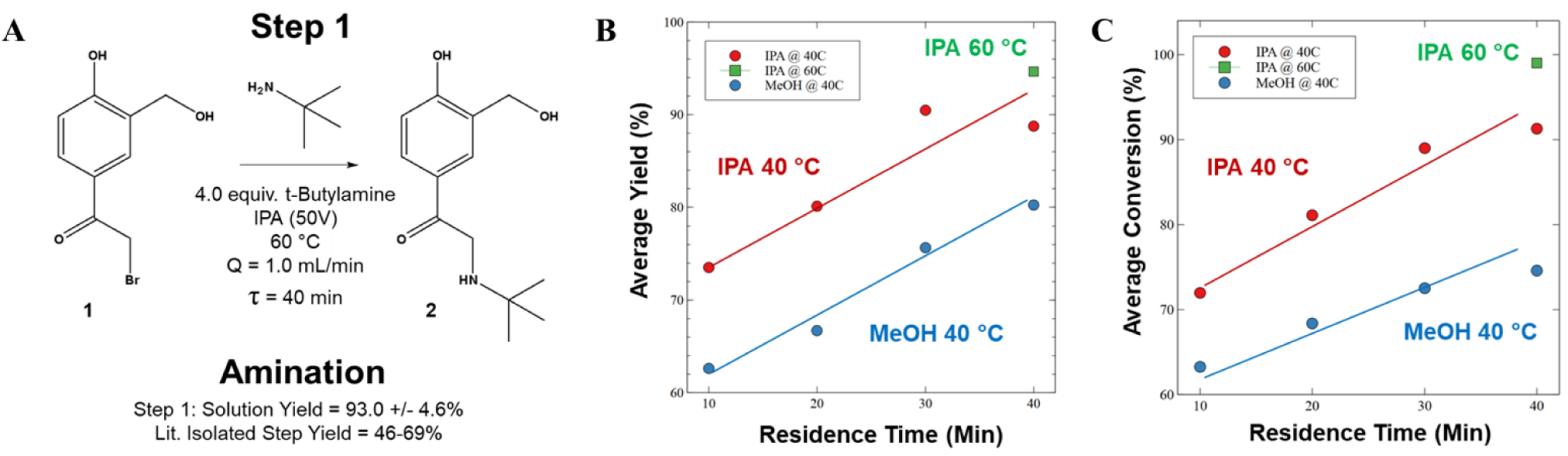
(A) Reaction Step #1, Amination; (B) the solution yield % of the intermediate molecule **2** after continuous LFD processing; and (C) conversion % of molecule **1** by HPLC analysis.

### Solids Filtration

During the amination reaction, four molar equivalents of TBA were added to the process stream for every molar equivalent of the bromo-diol SM. At ∼99% conversion, roughly one molar equivalent of TBA is consumed by the S_N_2 reaction and an HBr species is released. Meanwhile, a second equivalent functions as a weak Lewis base which scavenges the newly released HBr species to form a complexed TBA·HBr salt.^65^ The remaining two TBA equivalents exist as free amines which must be removed from the final process stream to meet purity specifications.

The precipitating TBA salts and oligomeric solids were removed via filtration. Filtration was manually performed during screening studies with 0.22 μm syringe filters, while continuous filtration was performed in flow using a Centramate tangential flow filter (TFF) from PALL Corporation, as configured to operate in a dead-end configuration. The TFF contained a Supor filter with pore sizes ranging from 0.22 to 0.65 μm as inserted between two stainless steel plates. Once the process stream was filtered, the intermediate material was added to a 500 mL round-bottom flask which served as a buffering vessel prior to distillation. See Turnage et al. for further information regarding the TFF filters.^67^

Any remaining TBA species are important downstream impurities to remove as it exists as an excess reactant in the initial amination reaction and as an impurity within the final API product. Additionally, TBA is difficult to monitor as it lacks molecular chromophores which absorb light above ∼200 nm, i.e., the lower limit for most UV detectors (see SI Figure S5). This makes TBA a challenging molecule to detect with UV-detectors during HPLC analysis. Thus, in-line distillation was investigated as a means of continuously removing the excess amine freebase while off-line GC-FID was used to monitor its removal.

### Continuous Distillation

A custom distillation system was developed for removal of the excess *tert*-butylamine freebase via continuous in-line distillation (see Figure 5). TBA possesses a boiling point of ∼46 °C whereas the next lowest boiling component consists of the process solvent, IPA (Bp^IPA^ ∼ 82 °C).^68^ This large temperature difference between TBA and IPA indicated that in-line distillation offered a continuous method of removing the excess free TBA from the process stream without suffering API losses traditionally associated with filtration and recrystallization. This process was first validated through a binary distillation study and transferred to continuous in-line distillation of the process stream.

**Figure 5:**
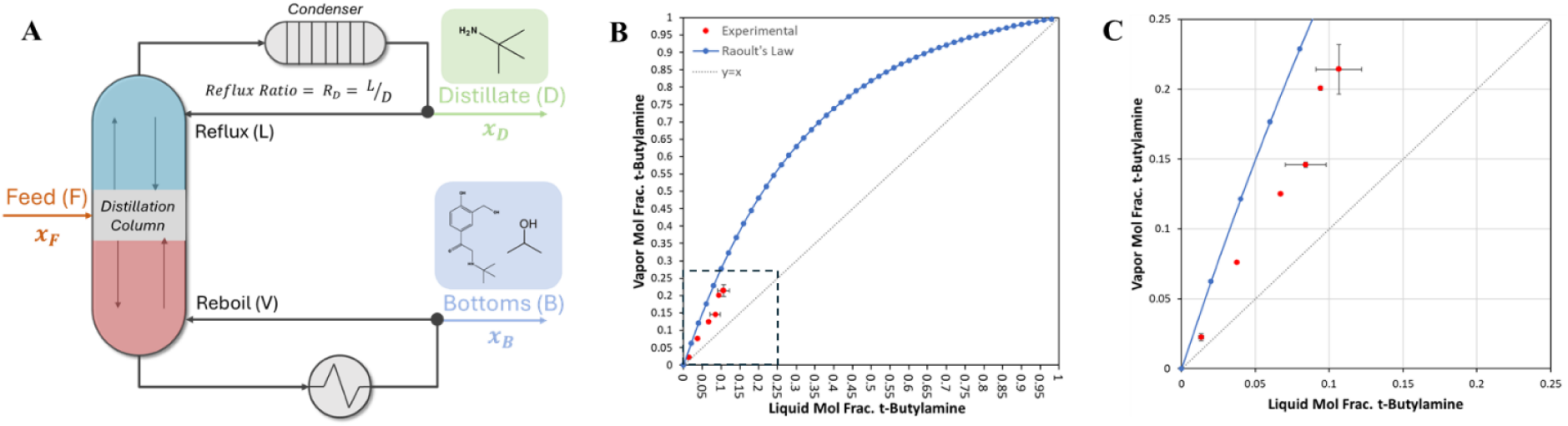
(A) Schematic diagram of the in-line distillation system; (B) a binary x–y diagram for a mixture of TBA and IPA as calculated by Raoult’s law (Blue) with experimental data in red; (C) expanded binary diagram in the lower left corner. Figure adapted in part with permission from ref 69. Copyright 2024 Justin T. Turnage.

A custom distillation column was assembled from a series of interconnected Hempel distilling columns, a 5.0 mL Dean–Stark tube equipped with an automated valve to enable removal of the distillate, and a three-necked round-bottom flask which served as the reboiler. A condenser was placed on top of the Dean–Stark tube and recirculating coolant was passed through the condenser (i.e., a 50:50 water and ethylene glycol mixture at 5 °C). The column was filled with aluminum packing (0.16″ ProPack from Xtractor Depot) to provide enough surface area to maintain multiple equilibrium contacts to enable separation across the column. The base of the column was heated with an enclosed heating mantel delivering a constant power supply of 7 W, effectively heating the bottoms to 92 °C. The column was encased in thermally insulating mineral wool to limit heat loss. Resistive temperature detectors (RTD’s) were installed along the reboiler, column, and condenser to monitor temperature. Finally, the entirety of the distillation column rests on a scale for weight monitoring and control within the system.

Binary distillation was previously simulated in ASPEN+ to confirm feasibility of TBA removal (see SI Table S2).^69^ During binary distillation, the bottoms product was periodically collected for analysis via gas chromatography to assess the composition leaving the column. Figure 5B–C shows experimentally obtained data (red) for a binary mixture of TBA and IPA at equilibrium, which nicely tracks the calculated vapor liquid equilibrium (VLE) curve (blue) as modeled with Raoult’s law. After validation of binary separation, the column was configured for continuous in-line distillation with an inlet feed line and outlet process stream.

During continuous distillation, product from the amination reactor was delivered to a round-bottom flask which was stirred and preheated on a stir plate. A precalibrated Ismatec Regloo peristaltic pump was assembled with 3-stop, 2.06 mm ID Viton tubing. The contents of the flask were pumped to the inlet of the distillation column at a rate of 3.0 mL/min. The condensed distillate was periodically removed by opening the valve at the top of the column to achieve a 1.0 reflux ratio (*R*_D_ = Reflux/Distillate). Thus, the valve was actuated to recycle the distillate stream back to the column every 6.0 s (i.e., reflux, *L*) followed by collection of the distillate (*D*) every 6.0 s.

Under continuous process conditions, the free amine entering the distillation column corresponds to a solution concentration of 8.0 mol % TBA in IPA. Whereas, during continuous distillation at 92 °C, the concentration of TBA in the distillate consists of 20 mol % TBA in IPA, while the bottoms product consists of 0.45 mol % TBA in process solvent. Thus, upon reaching steady state, the in-line distillation column enabled continuous removal of the free TBA species (i.e., ∼94% or 1.9 TBA equivalents) as assessed via GC-FID (see Figure S6). The resulting process stream was delivered to a holding vessel for subsequent hydrogenation to albuterol freebase within a catalytic reactor.

### Catalytic Reduction

A commercial Phoenix flow reactor from ThalesNano was utilized for reaction step two of the process, i.e., catalytic hydrogenation, in order to convert the intermediate **(2)** into albuterol freebase (see Figure 2, Molecule (3)). The liquid product exiting the distillation column was added to a 250 mL Erlenmeyer flask which served as a holding vessel prior to catalytic reduction. The solution in the holding vessel was then pumped to the catalytic reactor via a Knauer HPLC pump. Ultrahigh purity (UHP) hydrogen gas was supplied to the liquid process stream along a Y-shaped mixing junction. The mixing junction was equipped with a check valve to prevent H_2_ gas backflow. The reactor was configured with a 7.0 cm catalytic packed bed containing 0.25 ± 0.01 g of Pearlman’s catalyst, a commercial catalyst comprised of 20 wt % palladium hydroxide supported on carbon (i.e., Pd­(OH)_2_/C).^70,71^

A series of catalytic screening studies were performed in a design of experiment (DOE) fashion to assess the optimal liquid flow rate, concentration, pressure, and liquid weight hourly space velocity (L-WHSV) needed to achieve complete conversion of the intermediate **(2)** into albuterol freebase **(3)**. The catalyst readily enabled successful conversion of the intermediate into albuterol freebase at temperatures ranging from 25 to 80 °C and pressures ranging from 2 to 20 bar with negligible byproduct formation (see Figure 6).

**Figure 6:**
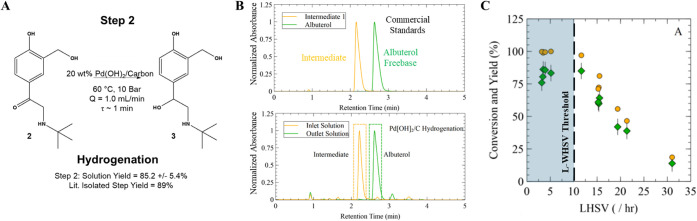
(A) Reaction Step #2, catalytic hydrogenation to form albuterol freebase; (B) representative HPLC chromatograms of commercial standards (Top) and the continuous hydrogenation reaction streams (Bottom) consisting of the SM solution (Yellow) and PBR product solution (Green) using 20 wt % Pd­(OH)_2_/C as a catalyst; and (C) conversion of the intermediate (%, Yellow) and solution yield (%, Green) of the albuterol freebase versus L-WHSV within the catalytic reactor.

The key species which enter the hydrogenation reactor include the solvent, the aminated intermediate, a dimerized SM impurity, and residual t-butylamine species. During hydrogenation, the intermediate’s ketone **(2)** is converted into a hydroxyl group resulting in the synthesis of albuterol freebase **(3)** as shown in Figure 6B. Similarly, the minor residual dimer impurity was also observed to reduce over the catalyst to form a hydrogenated dimer weighing ∼4 amu heavier than the initial dimerized species. Additionally, the dimer was also observed to crack apart over the catalyst to form a decomposed impurity and one equivalent of albuterol. These species were each identified via LC-MS and quantified with HPLC (see SI Figures S7–S8).

An optimal conversion (99.5 ± 0.1%, 95% CI), solution yield (85.2 ± 5.4%, at 95% CI), and throughput was obtained at a temperature of 60 °C, a pressure of 10 bar, and a 1.2 mL/min flow rate. Minimal variations were observed in both yield and conversion when altering the temperature and pressure of the reactor within the instrument’s available operating range. Similarly, minimal effects were observed when altering the gas flow rate. However, conversion and solution yield were both observed to diminish as the flow rate of the process stream was increased above a critical threshold (see Figure 6C). Complete conversion and optimal yields were observed when L-WHSV ≤ 10 [h^–1^]; while reduced conversion and solution yield were observed with L-WHSV > 10 [h^–1^]. Here L-WHSV is defined as the liquid flow rate (*Q*_L_) multiplied by the intermediate concentration (*C*_int_) per gram of catalyst (*m*_cat_):

1 $$L-WHSV=Q_{L}\times \frac{C_{Int}}{m_{cat}}$$

This data demonstrates that L-WHSV is the key factor necessary for successful hydrogenation of the intermediate **(2)** to form albuterol freebase **(3)**. When exceeding the L-WHSV threshold, the intermediate molecules do not have enough active sites and residence time to effectively hydrogenate over the catalyst. Conversely, when maintaining a flow rate of the process stream such that L-WHSV ≤ 10 [hr^–1^] per gram of catalyst, the system is provided with enough residence time to complete the reaction. Thus, L-WHSV is a key parameter enabling scale-up of the system. See Kay et al. for further catalytic hydrogenation discusion.^72^

### API Salt Formation

During the third processing step of the continuous system (see Figure 3), the albuterol freebase **(3)** was precipitated via sulfation with 0.5 mol equiv to form the API, albuterol sulfate. After hydrogenation, the albuterol freebase process stream was pumped into an RBF at a rate of ∼1.2 mL/min. Next, a solution of 0.75 M H_2_SO_4_ in IPA was added dropwise to the CSTR at a rate of 25 μL/min. The reaction mixture was subsequently removed from the API salt vessel at a rate of 1.23 mL/min providing a residence time of ∼40 min. Samples were collected along the CSTR outlet for pH, turbidity, and HPLC analysis.

This continuous unit operation was accomplished by converting an IKA stir plate and a 100 mL three-necked RBF into a CSTR, while continuous addition of H_2_SO_4_ was delivered with a single channel IPS-12 syringe pump. The sample was stirred at 25 °C for various residence times while off-line UV–vis spectroscopy was performed to assess precipitation via turbidity analysis during startup (see Figure 7B–C). Samples were collected at multiples of τ ranging from 0 to 6 and analyzed at λ = 632 nm with the spectrometer. The samples initially appeared clear (τ ∼ 1), indicating minimal precipitation. However, after running the CSTR for an extended time (τ > 4; 160 min), the transmittance quickly diminished and the solution became opaque, indicating the precipitation of albuterol sulfate and the formation of crystals. Thus, an overall sulfation CSTR startup of ∼3 h enabled adequate time for initial sulfation and precipitation of the API.

**Figure 7:**
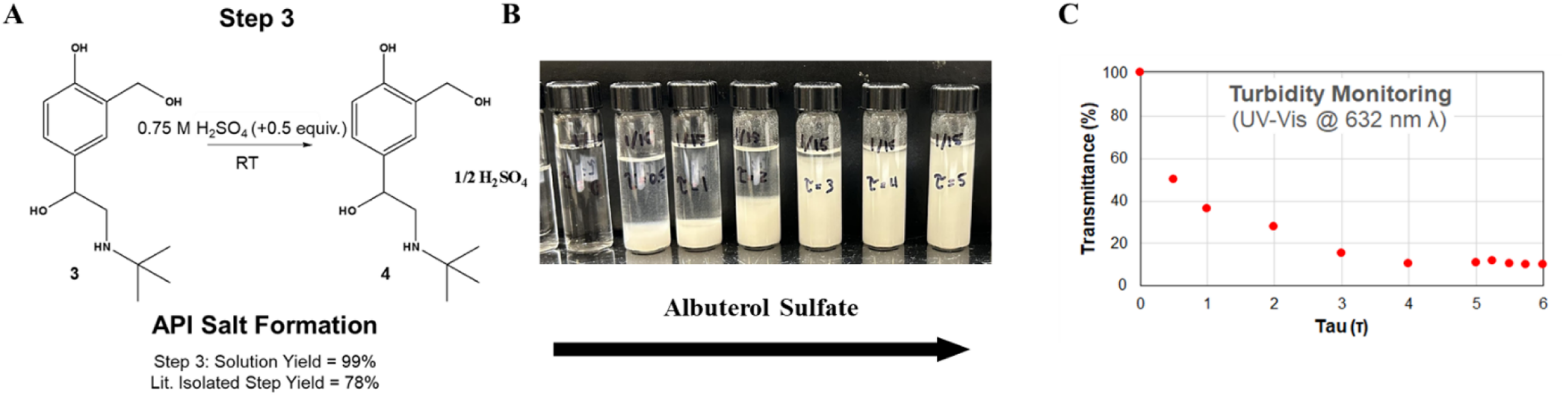
(A) Reaction Step #3, continuous sulfation to form the final API, albuterol sulfate; (B) precipitated albuterol sulfate solutions during startup, (C) UV–vis transmittance (%) confirming precipitation of suspended albuterol sulfate particles.

The final API slurry was collected and purified via Büchner funnel filtration and recrystallization. The slurry was filtered over 0.2 μm filter paper and rinsed with IPA under house vacuum. This process was repeated 3–5 times and followed by recrystallization in IPA to remove the remaining impurities. The product was then analyzed with orthogonal off-line NMR, HPLC, and LC-MS characterization. This process achieved an isolated step yield of 76.8% and +95% purity.

### Online ^1^H NMR Analysis

A Magritek 80 MHz Spinsolve NMR spectrometer was utilized to conduct in-line NMR analysis to validate the structure of as-synthesized species and to assess future integration of ^1^H NMR as an in-line PAT in a scaled-up manufacturing system (see Figure 8). A key series of protons were identified which served as a molecular fingerprint for chemical identification and quantification. This enables direct analytical process monitoring of crude reaction mixtures via ^1^H NMR without intermittent purification steps. ^1^H NMR analysis was performed on the bromo-diol SM **(1)**, the isolated intermediate **(2)**, the albuterol freebase **(3)**, and albuterol sulfate **(4)**.

**Figure 8:**
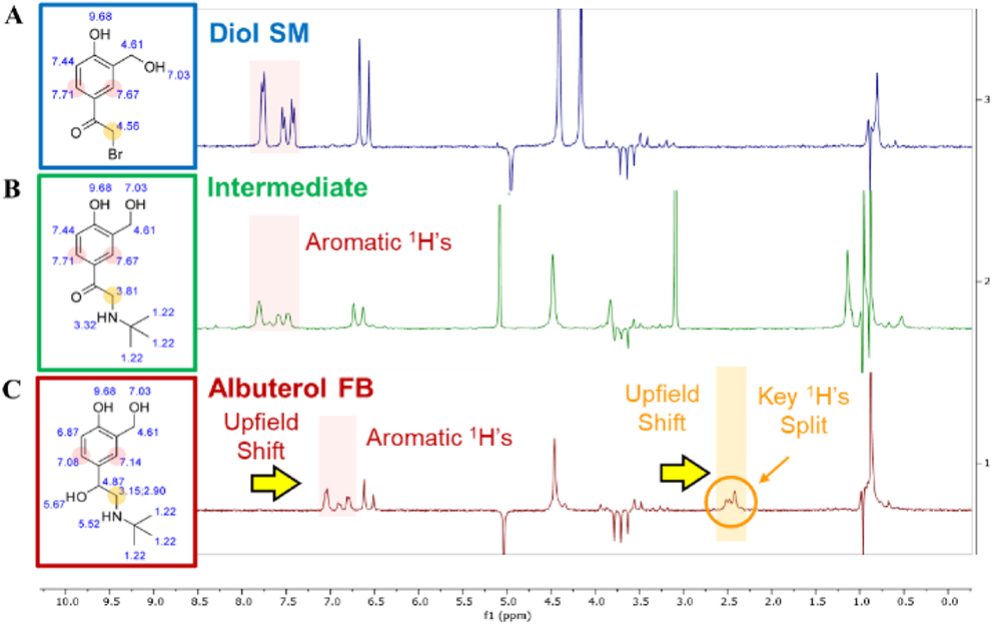
^1^H NMR spectra of major albuterol-related species as measured with the 80 MHz Magritek Spinsolve NMR. The ^1^H NMR peak simulations are presented (Left) along with the corresponding ^1^H NMR spectra (Right) for the key molecular species including (A) the SM in Blue; (B) the intermediate in green; and (C) the albuterol freebase in red. Chemical standards were prepared at concentrations ranging from 1.0 to 50 mg/mL in IPA with a 30 s scan time. Wet suppression mode was utilized to suppress the IPA solvent peaks at ∼5.6 ppm, 3.5–4.0 ppm and ∼1.0 ppm.

Key protons for each chemical species were identified. ChemDraw was utilized to simulate the general ^1^H NMR peak positions for each proton in the synthetic pathway (see SI Figure S9). As is typical with ^1^H NMR, protons on the aromatic ring were predicted to resonate downfield at ∼7–8 ppm; protons on −CH_2_– alkanes were located midfield ∼3–5 ppm, and methyl protons were located upfield ∼1.2 ppm. A key pair of geminal protons were identified, these protons are located between the ketone and bromine on the bromo-diol starting material (see SI Figure S10). The simulation suggests that as the reaction progresses, the key ^1^H NMR peak shifts upfield from ∼4.5 ppm (i.e., starting material), to ∼3.8 ppm (i.e., intermediate), to ∼3.0 ppm (i.e., albuterol freebase). Upon hydrogenation of the ketone group to form albuterol, vicinal protonation leads to proton coupling and peak splitting of the geminal protons at 3.15 and 2.90 ppm. Lastly, it should be noted the simulated predictions are approximations based on theoretical peak positions; thus, some variations in peak location may be observed due to the use of IPA as a solvent.

After simulating the proton peak locations, as-synthesized chemical standards were prepared in IPA and analyzed with a Magritek 80 MHz Spinsolve NMR. Indeed, the diol starting material’s key geminal protons, located between the ketone and bromine group, were observed to shift upfield upon amination as observed when comparing Figure 8A–C. As predicted by the simulation, upon hydrogenation of the ketone, the geminal proton peak shifted upfield and coupled with the new vicinal hydrogen; this coupling caused the geminal ^1^H peak to split. This is highlighted by the orange shaded region in Figure 8. These geminal protons are key protons as they directly neighbor the two locations in which major reaction steps proceed. Thus, these protons and their corresponding NMR peaks are unique features which enable the identification and quantification of key molecular species in the synthetic pathway.

Having identified the location of each key NMR proton peak, a systematic study was performed to calibrate chemometric models which would enable quantification of the concentration of each key molecular species via in-line ^1^H NMR. To accomplish this, standards for each key species (i.e., Diol-SM, the intermediate, albuterol freebase, and TBA) were prepared and diluted in IPA at concentrations ranging from 1.0 to 150.0 mg/mL. The samples were analyzed using 30 s scans. The ^1^H NMR peaks corresponding to the key geminal protons in Figure 8 were integrated and a linear regression methodology was applied in order to obtain quantitative models which relate the ^1^H NMR peak area to each analyte’s concentration (see SI Figure S11). Next, linear regression was performed to produce chemometric calibration curves for each species using JMP software. Finally, 95% confidence intervals (CI) were determined for each regression line corresponding to each sample.

These powerful quantitative models demonstrate a linear trend which enables the detection of each molecular species and a key impurity (i.e., TBA) within the synthetic process stream via engineering of in-line sampling within the final end-to-end system (see Discussion). Additionally, several handles exist which can be used to improve these calibrated models. If more resolution is required, longer scans can be conducted at the trade-off of sampling frequency to enable improved limits of detection (i.e., LOD) for impurities such as t-butylamine. This area of research is currently under investigation for integration of in-line ^1^H NMR within the scaled-up pilot plant.

### Discussion: Scale-up for a Candidate AMT Pilot Plant

This work encompasses our early conceptual research and development to engineer a candidate AMT system for the synthesis of albuterol sulfate in liquid dosage form via a continuous API manufacturing system. The prototype laboratory system detailed within this text was assembled from commercial flow chemistry reactors and in-house solutions to achieve a multistep telescoped process for the synthesis of albuterol sulfate. The prototype system now serves as a model for the ongoing development of a larger pilot plant system for production under current good manufacturing processes (cGMP). This emerging system serves as a potential AMT candidate as it constitutes a novel technology that “*increase[s] or maintain[s] the supply of a drug ··· that is on the drug shortage list under section 506E of the FD&C ACT (21 U.S.C. 356e).*”^7^

To begin the engineering process, a series of batch chemistry studies were performed to screen synthetic chemistry routes based on their potential for seamless transfer to a continuous manufacturing system. These individual transformations were then utilized within standalone continuous flow unit operations to optimize the process conditions necessary for advanced manufacturing. Finally, each unit operation was integrated into telescoped systems to demonstrate the process on a laboratory scale. The resulting design can be readily scaled up to increase throughput for the production of albuterol sulfate on an industrial scale. The system will be integrated in an end-to-end fashion with a second candidate AMT system currently under development by researchers at the Center for Supported Organic Particulate Systems (i.e., C-SOPS). The integrated system will enable drug synthesis and packaging of the final drug product within sterile vials.^17^

During the initial planning phase of the continuous API manufacturing system, legacy off-patent batch process chemistry routes involving salicylaldehyde were examined for the synthesis of albuterol sulfate. Next, a series of batch chemistry experiments were conducted to assess route feasibility. The primary goal of the initial batch screening studies was to eliminate as many processing steps as possible which involved freebasing, solids formation, and powder handling while selecting a new chemistry route which would seamlessly transfer to a continuous flow system. These early synthetic studies enabled the streamlined development of a new process chemistry route with limited potential for clogging or the need to incorporate solid-handling process steps. This ultimately led to the development of a new synthetic pathway for albuterol sulfate conducive to AMT manufacturing in flow.

The system described herein incorporates a series of mixing vessels comprised of stir plates and RBF's which contain the reactants in IPA under inert atmosphere to prevent degradation of the intermediate species. The reagents are first pumped to a laminar flow reactor (Vaportec E-Series Reactor) via a peristaltic pump at 1.0 mL/min. The bromo-diol starting material **(1)** then undergoes an S_N_2 reaction with four equivalents of TBA to form a key intermediate species **(2)**. This reaction is readily accomplished within the LFR at 60 °C and a residence time of 40 min to achieve a 93.0 ± 4.6% solution yield of molecule **(2)** and 99.0 ± 0.79% conversion of the SM. For successful scale up of the pilot plant, it is recommended to utilize at least 1/8–1/4″ ID tubing and larger fittings to limit the potential for clogging, as solids tend to clog narrow connections <1/16″ ID. Residual solids are removed from the process stream along a dead-end polishing filter (i.e., Pall Corp.) located downstream from the LFR.

Next, an in-line distillation column was utilized to remove the excess TBA equivalents at a 1.0 reflux ratio when heated to the boiling point of the process stream (92 °C). TBA is difficult to detect with HPLC UV-DAD detectors as it lacks a chromophore and undergoes a solvatochromatic shift as the solution pH is varied (λ_abs_ < 200 nm). However, the large difference in boiling points between TBA (Bp ∼ 46 °C) and IPA (Bp ∼ 82 °C) enables removal of the more volatile free TBA species via distillation while retaining the intermediate **(2)** in the bottoms of the column. Additionally, in-line distillation enabled the removal of TBA without the downstream yield losses commonly associated with filtration and recrystallization. Additional makeup IPA is supplied to the process stream along a buffer tank downstream from the distillation column to maintain the system basis.

A catalytic packed bed reactor (ThalesNano Phoenix Reactor) enabled reduction of the intermediate **(2)** to form albuterol freebase **(3)** by reacting UHP H_2_ over 0.25 g of 20 wt % Pd­(OH)_2_/Carbon catalyst. This is achieved for a 1.0 mL/min basis at 60 °C, 10 bar pressure, and an L-WHSV < 10 [h^–1^] to achieve a 99.5 ± 0.1% conversion and 85.2 ± 5.4% solution yield. A key parameter for catalytic hydrogenation of the intermediate is the liquid weight hourly space velocity. By maintaining a L-WHSV below the critical threshold (i.e., 10/h), the process stream has enough residence time and active catalyst sites to fully convert to albuterol freebase. Conversely, when exceeding the L-WHSV, reduced conversion and yield will occur as the intermediate does not have the necessary residence time to fully react. Reactor scale up is enabled by using more granular versions of Pearlman’s catalyst (*D*_50_ = 150–190 μm) in larger reactors with increased catalyst loadings ranging from 3 to 5 g (see Methods). Granular catalysts with larger particle sizes help prevent pressure drop across the reactor, while the larger reactor provides increased catalyst loading to enable higher flow rates and ultimately increased throughput (see SI).

The final transformation, API precipitation via sulfation, is achieved in a CSTR style reactor downstream from the hydrogenation reactor. The effluent from the catalytic reactor is sent to the CSTR via a peristaltic pump where it is mixed with 0.75 M H_2_SO_4_ in IPA as delivered through a syringe pump. The flow rate of the acid solution is scaled to deliver 0.5 equiv of sulfuric acid per mole of API. A second channel on the peristaltic pump is utilized to pump the unpurified API product slurry from the CSTR to a final API holding tank to await collection. While additional purification is needed, the final isolated step yield after Büchner funnel purification, recrystallization, and drying was 76.8% (i.e., isolated step yield). This correlates to an overall process yield from Step 1 to Step 3 with final purification of 60.2% (Final Isolated Product Yield). The solution yields in this process exceed the isolated yields reported in the literature for similar legacy batch reaction pathways and are achieved without the need for isolation between each synthetic step.^58^

The current laboratory scale system is capable of producing albuterol sulfate at a rate of +2,000 mg/h when operating at a 1.0 mL/min basis and an SM concentration of 50 mg/mL. A standard liquid albuterol sulfate dose formulation consists of 3.0 mg of API within a 3.0 mL aqueous solution.^48^ Thus, this throughput basis is enough API by mass to produce +700 liquid doses per hour along the downstream outlet of the CSTR sulfation unit. The overall theoretical throughput is provided by Table 1. The system outlined above is projected to produce 1.0 kg of API if operated for and extended 15-day campaign (see SI Table S6).

**Table 1:** Stream Table and Theoretical Production Rate for Unit Operations in the Continuous System (Basis = 1.0 mL/min)^a^,^b^

System basis: 1.0 mL/min	Unit Op. One Mixing	Unit Op. Two Amination	Unit Op. Three Distillation	Unit Op. Four Reduction	Unit Op. Five Sulfation	Unit Op. Six Purification
**Mass Flow Rate** ^**(Species)**^ (mg/Hour)	3,000^(SM)^	2,707^(INT)^	2,707^(INT)^	2,321^(FB)^	2,769^(API)^	2,127^(API)^
**Molar Flow Rate** ^**(Species)**^ (mM/Hour)	12.2^(SM)^	11.4^(INT)^	11.4^(INT)^	9.70^(FB)^	9.60^(API)^	7.37^(API)^
**Step Conversion** (%)	-	99.0%	-	99.8%	99.0%	99.9%
**Step Solution Yield** (%)	-	93.0%	-	85.2%	99.0%	76.8%^(Iso)^
**Overall Solution Yield** (%)	-	93.0%	-	79.2%	78.4%	60.2%^(Iso)^
**Theoretical Dose Rate** (Dose/Hour)	1,177	1,094	1,094	938	923	709

a **Formulation:** A dose is defined in this text as 3.0 mg of API within a 3.0 mL solution (i.e., [API] = 1.0 mg/mL).

b **Abbreviations:** Starting Material (SM), Intermediate (INT), Albuterol Freebase (FB), Albuterol Sulfate (API), Isolated (Iso).

The conceptual AMT process described above is currently being scaled into a larger pilot plant for the continuous manufacturing of albuterol sulfate. This candidate AMT platform implements a series of new automation solutions for end-to-end drug manufacturing which enables synthesis of the API and encapsulation within labeled vials all within a single standalone plant. To achieve this, the platform integrates online ^1^H NMR for PAT analysis of the process stream, automated distillation reflux control valves, a series of standard PAT’s (i.e., pressure transducers, thermocouples, flow meters, etc.), and an automated Nutsche-style laboratory filter dryer for automated purification. The final system fits within a facility the size of a shipping container (8.0′’ × 8.5′ × 40′).

A preliminary economic analysis was conducted to assess the cost of goods (COG) manufactured within the continuous flow system. The current market price for albuterol sulfate is approximately ∼$146 per kg,^73^ whereas the market price for the bromo-diol SM is approximately $490 per kg as obtained from our suppliers (e.g., Ambeed). The market for the SM is limited to research-scale quantities and corresponds to a comparatively higher price versus the API. Thus, in order to improve the economic feasibility of the process, the team has investigated additional upstream processing steps to produce the bromo-diol SM from cheaper salicylaldehyde materials (see SI Figure S13). We plan to report our findings in future publications.

The candidate AMT system incorporates automated PAT’s (i.e., P, T, Q) to analyze critical process parameters (CPP) while ensuring the API meets critical quality attributes (CQA). Purity is assessed by online ^1^H NMR spectroscopy. NMR spectroscopy is a powerful analytical tool that offers unsurpassed structural information about the molecules contained within a liquid sample. ^1^H NMR spectra can now be obtained on the order of a few seconds to minutes depending on settings and the concentration in the system. The number of nuclei within the NMR provides a direct correlation between the spectral peak area and species concentration; a key concept which enables quantification of chemical concentration. Additionally, NMR is able to quantify species which lack a chromophore (e.g., TBA).

Until recently, online NMR was not possible as most traditional NMR systems utilize strong magnetic fields and liquid N_2_ which necessitate bulky dewars and a dedicated room to house the instrument. Recently however, smaller benchtop ^1^H NMR systems have come onto the market. These systems have a sample channel running through the instrument and insertable glass flow cells which enable users to pump chemical process streams through the NMR. By pausing the flow for a few minutes, the NMR can then scan the process stream to obtain online NMR data. A key feature of the pilot plant system is the incorporation of ^1^H NMR as a direct online PAT to validate the API’s chemical structure and product composition throughout the system. This is accomplished by multiplexing an 80 MHz Magritek Spinsolve NMR to sample the process stream at upstream and downstream locations within the system (see SI Figure S12). In this work, the team completed the early structural analysis of the related albuterol species (see Figure 8) via NMR spectroscopy and completed the necessary calibration curves to incorporate online NMR within the proposed AMT system.

The primary factors leading to the selection of a benchtop ^1^H NMR for online PAT integration was its ability to (I) systematically identify the protons on each molecular species in the synthetic pathway; (II) detect chemical standards of increasing concentration; (III) correlate integrated NMR peak data to concentrations as assessed with HPLC; and (IV) calibrate ^1^H NMR chemometric models. This emerging AMT candidate system is being constructed to enable real-time quality control (RTQC) of the automated manufacturing system. Additionally, the online NMR system is equipped to periodically analyze the downstream product which exits the filter dryer to provide a final quality control check for impurities before sending the product to drug product packaging.

Final API purification is completed in the pilot plant via an automated Nutsche filter dryer. This is accomplished via a series of rinsing cycles with IPA followed by a final redissolution in water to form the liquid dose form (i.e., 3.0 mg of albuterol sulfate within a 3.0 mL aqueous solution). The filter dryer cycles enable the removal of any remaining TBA·HBr salts, starting material oligomers, and decomposed species as was demonstrated via manual filtration on a smaller lab scale. Lastly, a final redissolution with purified water for injectables enables dilution of the final API to produce a formulation which meets ICH purity standards.^14^

The pilot plant incorporates system-wide process control architectures using a distributed control system (DCS). DCS’s enable live monitoring of CPP’s across each unit operation within the manufacturing system and enable feedback control to correct for CPP disturbances. DCS process control is achieved by utilizing computerized control loops and autonomous controllers to maintain CPP’s within critical thresholds (i.e., normal operating range, NOR). Additionally, DCS enables acceptance or rejection of chemical process streams prior to packaging based on product purty. The DCS system is directly coupled with the online PAT’s to monitor live process data for direct comparison against a Digital-Twin model. Lastly, all PAT data is recorded via a data historian to ensure the process meets current good manufacturing practices.

## Conclusions

AMT’s offer new avenues toward continuous API process intensification that were previously unavailable via batch processing. This includes the reduction of waste, the incorporation of heterogeneous catalysts, less hands-on processing, and real-time quality control. Additionally, AMT solutions offer continuous API production to meet high throughput targets with increased safety measures. Finally, the incorporation of online PAT’s offers the ability to remotely monitor API synthesis from RSM’s to API drug products while using online spectroscopic PAT’s to assess CPP’s all within a single streamlined manufacturing process. This unique strategy can ensure product quality while meeting FDA and ICH standards for drug product purity.

The system detailed in this text enables the production of +2,000 mg/h of albuterol sulfate, a bronchodilator asthma drug on the FDA’s drug shortage list. The overall process can generate +700 doses per hour at a 1.0 mL/min basis for use in a liquid dose albuterol sulfate drug formulation (i.e., 3.0 mg in a 3.0 mL aqueous solution). This system can reach an overall solution yield of 78.4% with a final isolated yield of 60.2%, values which meet and exceed those reported elsewhere in literature. Additionally, we have demonstrated ^1^H NMR’s ability to analyze the key chemical species involved in the process. The team is currently building a larger pilot plant system for continuous albuterol sulfate manufacturing with online ^1^H NMR analysis; and will apply for both AMT and ANDA applications at the conclusion of the project.

The emergence of continuous AMT systems has poised the U.S. pharmaceutical manufacturing industry for a resurgence in growth in generic drug production. It is with this evolving manufacturing strategy and the solutions discussed in this text, that the U.S. can restructure the global RSM and API supply chain. The result of this field of work will help foster future AMT development, help reduce drug prices, and eliminate drug shortages for key lifesaving drugs.

## Chemicals

The starting material, i.e., 2-Bromo-1-[4-hydroxy-3-(hydroxymethyl)­phenyl]­ethan-1-one (Species (1), Figure 2, CAS# 62932-94-9, purity >95%) was obtained from Ambeed and used as a SM for the synthesis of the albuterol intermediate (Figure 2, Molecule 2, CAS# 156547-62-5). An intermediate standard **(2)** was isolated from the batch synthetic studies via precipitation with HCl, Büchner funnel filtration with IPA (i.e., rinsing with 3x cake volume) and recrystallization in IPA. Chloride content was quantified via Mohr’s titration with AgNO_3_. The isolated intermediate, Molecule 2, was compared with a commercial standard purchased from Clearsynth (1.0 g, purity >95%). The as-synthesized albuterol sulfate **(4)** was purified via filtration with IPA using a Büchner funnel and 0.2 μm Nylon filter paper. A commercial albuterol sulfate standard was purchased from Glentham Life Sciences (purity ≥98.0%) for confirmation with the as-synthesized material. Tert-butylamine (CAS# 75-64-9) was purchased from Oakwood Chemicals (500 g, purity >95%). Dry methanol was purchased from Fisher Chemical (HPLC grade, purity >99%); extra dry isopropanol (IPA) was purchased from Fisher Chemical (ACS grade, purity >99%). Prior to experimentation, the IPA was dehydrated using molecular sieves from Fisher (Grade 514, Type 4A, 8–12 Mesh Beads). IPA dehydration with molecular sieves reduced the water content to 0.016 ± 0.001% as measured by Karl Fischer Titration (Mettler Toledo C10s).

## Methods and Instrumentation

### Vaportec E-Series Reactor: S_N_2 Amination In-Flow

The S_N_2 amination reaction was configured for continuous operation using a Vaportec E-Series LFR reactor equipped with a 20 mL tubular reactor vessel comprised of 1/16 in. ID tubing. The solutions were pumped from their respective flasks through 2.06 mm ID Viton tubing via a four-channel Ismatec Reglo peristaltic pump at 1.0 mL per min. The peristaltic pump was precalibrated using the appropriate process solvent (i.e., MeOH or IPA) during the initial screening studies. The reactants were delivered to the reactor with a peristaltic pump and mixed along a T-junction comprised of PEEK fittings. The combined reactant stream was then fed through the tubular reactor. The residence time (τ) was systematically varied from 3.7 to 60 min by tuning the combined reactant flow rate from 0.3 to 10 mL/min. The reaction was screened at 25, 40, and 60 °C. The first reactant vessel contained the SM **(1)** in process solvent at a concentration of 100 mg/mL. The SM vessel was preheated at 40 °C prior to LFR delivery. The second reactant vessel contained t-butylamine in process solvent at a concentration of 120 mg/mL. The concentrations of each reagent were selected to enable equal flow rates of each stream while maintaining a 4:1 molar ratio (i.e., SM:t-butylamine). The reactor tubing was coiled around a spool and encased within a glass housing provided by Vaportec. The reactor was heated with flowing air at temperatures ranging from 25 to 60 °C. During screening studies, analytical samples were collected along the outlet, manually filtered with a 0.22 μm syringe filter, and analyzed offline with HPLC and NMR.

### Continuous Distillation

A custom in-line distillation column was assembled using common laboratory glassware. A 250 mL three-necked round-bottom flask served as the reboiling unit and was connected to a series of three Hemple 24/40 joint distilling columns. The column was filled with 0.16” ProPack aluminum packing (Xtractor Depot). A 5.0 mL Dean–Stark tube was placed atop the column. All joints were sealed with Molykote high vacuum grease (Dupont). A condenser unit was fitted above the Dean–Stark tube while flowing chilled water (5 °C) was fed by a Julabo recirculating chiller. The base of the column was heated with an enclosed heating mantel at 82 °C for binary distillation, and 92 °C for continuous in-line distillation. The column was insulated with mineral wool. K-type thermocouples were installed along the reboiler and the condenser. Additional RTD’s were installed vertically along the column under the insulation. The chemical product from the amination reaction was stored in a round-bottom flask and preheated on a stir plate. During operation of the column, the process stream was delivered to the top of the column using a precalibrated Ismatec Regloo peristaltic pump as equipped with 3-stop, 2.06 mm ID Viton tubing. The contents of the flask were pumped to the inlet of the column at a rate of 3.0 mL/min. The reflux ratio (*R*_D_) is defined as the volumetric ratio of reflux (L) to distillate (D). This ratio was set to *R*_D_ = 1.0 and controlled by directing aliquots of condensate along the Dean–Stark tube in 6.0 s intervals. After each interval the valve was manually opened to reflux the aliquot or collect the distillate. The bottoms flow rate was dictated by weight monitoring and control of the column. The average flow rate of the bottoms product stream was 1.5 mL/min

### Thalesnano Phoenix Reactor: Catalytic Hydrogenation

Catalytic hydrogenation screening reactions were conducted using a commercial Phoenix Flow Reactor from ThalesNano. To summarize, the hydrogenation reactions were performed over Pearlman’s catalyst as purchased from Sigma-Aldrich (i.e., 20 wt % palladium hydroxide on carbon (Pd­(OH)_2_/C); CAS# 12135-22-7). The catalyst was packed into a 70 mm packed bed reactor cartridge with a 64 mm internal length and a 4.0 mm ID. The reactor was packed with 0.25 ± 0.01 g of catalyst and sealed with a mesh frit and O-ring. Liquid reaction solutions containing the filtered intermediate (Molecule 2) were delivered to the reactor via a Knauer HPLC pump at flow rates ranging from 0.3 to 2.4 mL per minute. The pump was precalibrated to ensure proper reagent flow rate. UHP H_2_ gas was mixed with the inlet stream along a Y-junction at 5–30 mL/min. The reactor pressure was controlled using a back pressure regulator and set at pressures which ranged from 0 to 20 bar. Temperatures were screened from 25 to 80 °C. Reactor scale up tests were conducted using a large ThalesNano MMS Reactor with *L* = 250 mm and ID = 9.4 mm to enable increased catalyst loading (*i.e*., 3–5 g). During reactor scale up more granular versions of Pearlman’s catalyst (i.e., Evonik, Noblyst F1612, D_50_ = 150–190 μm) were utilized to prevent pressure drop across the reactor at high flow rates, Q > 3.0 mL/min (see SI Figure S14).

### CSTR: Continuous Sulfation in-Flow

The albuterol freebase (CAS# 18559-94-9) was precipitated via sulfation with 0.5 mol equiv of H_2_SO_4_ (CAS# 7664-93-9) to form albuterol sulfate. The albuterol freebase process stream was pumped into a 100 mL three-necked RBF at a rate of ∼1.2 mL/min. Next, a solution of 0.75 M H_2_SO_4_ in IPA was added dropwise to the CSTR at a rate of 25 μL/min. The reaction mixture was subsequently removed from the sulfation vessel at a rate of 1.23 mL/min providing a residence time of ∼40 min. An IKA stir plate and a 100 mL three-necked round-bottom flask (RBF) were converted into a CSTR, while continuous addition of H_2_SO_4_ was delivered with an IPS-12 single channel syringe pump. The sample was stirred at 25 °C and pumped to a round-bottom flask for collection. The final API was collected and purified during the initial screening studies via filtration and rinsing with IPA through a Büchner filter under house vacuum followed by recrystallization to remove the remaining impurities. The product was then analyzed with orthogonal off-line NMR, HPLC, and LC-MS characterization.

### High-Performance Liquid Chromatography (HPLC)

High performance liquid chromatography was utilized by employing an Agilent 1200 series HPLC equipped with an Eclipse XDB-C18 column (5.0 m; 4.6 μm × 250 mm). Samples were prepared for HPLC analysis by filtering, massing, and diluting the sample with methanol in a volumetric flask to achieve a target concentration of 1–4 mg/mL. Calibration curves were prepared for each primary chemical species involved in the reaction pathway using commercial standards. Peak integration was performed and compared to the calibration curve for accurate chemical assay. The chemical species were analyzed using two separate HPLC methods, each of which employed mobile phase gradients consisting of 0.1% phosphoric acid in water and pure methanol (HPLC grade). Method one was developed to assess the chemical composition of the S_N_2 amination reaction while the second method was utilized to assess the chemical composition of the hydrogenation reaction and downstream products. The first method utilized an initial mobile phase consisting of 85% H_3_PO_4_ buffer solution and 15% MeOH at a flow rate of 1.7 mL/min and a column temperature of 30 °C. The second method utilized an initial mobile phase consisting of 95% H_3_PO_4_ buffer solution and 5% MeOH at a flow rate of 1.5 mL/min and a column temperature of 30 °C. A diode array detector (DAD) scanned the eluting HPLC samples at a wavelength (λ) of 220 nm.

### Liquid Chromatography Mass Spectrometry (LC-MS)

Liquid chromatography mass spectrometry was utilized to assess the impurity profile of each reaction involved in the albuterol sulfate synthesis pathway. LC-MS was conducted using an Agilent 1200 Infinity Lab System which was equipped with a Zorbax SB-C18 column (3.5 μm; 2.1 × 150 mm), a DAD, and an MS detector. The DAD assessed chemical absorbance to determine the relative composition of major impurities (i.e., dimers and trimers) while the MS detector analyzed the mass-to-charge ratio (*m*/*z*) of each species as they eluted through the system.

### Gas Chromatography Flame Ionization (GC-FID)

Gas chromatography was performed using an Agilent 7000 series GC equipped with a flame ionization detector (FID). A Restek Rtx-Volatile Amine column (L 30 m x ID 320 μm × FT 5 μm) was installed on the system. Helium (UHP grade) was utilized as the carrier gas with a flow rate of 2.0 mL/min and a 10:1 split ratio. The oven temperature was ramped from 60 to 70 °C at 2 °C/min. GC-FID calibration curves were prepared by mixing IPA and tert-butylamine standards in acetonitrile (ACN) at concentrations ranging from 0.5 to 5.0 mg/mL. Injections for calibration curves were made in triplicate. During analysis of distillation samples, the sample was diluted in ACN and injected into the GC for analysis while concentration was assessed with the calibration curve.

### UV–vis Spectroscopy

UV–vis spectroscopy was performed with an Agilent, Cary 5000 spectrometer to assess the absorbance spectra of key molecular species. Additionally, UV–vis was used to assess the precipitation of albuterol sulfate samples (% Transmittance) during continuous precipitation. During analysis, samples were placed in 1.0 cm quartz cuvettes and analyzed at λ ranging from 200 to 750 nm. During precipitation studies, a wavelength of 632 nm (i.e., the middle of the visible spectrum) was used to assess precipitation of the API.

### ^1^H Nuclear Magnetic Resonance Spectroscopy

Proton NMR was conducted using a Magritek 80 MHz benchtop NMR. Samples were placed in glass NMR tubes and analyzed in 1D ^1^H mode, ^13^C satellite decoupling mode (i.e., 1D ^1^H­{^13^C}), and under solvent suppression mode. Optimal resolution was observed when utilizing a 3.2 s acquisition time, a 7–30 s repetition time, and scans ranging from 2 to 16. The data was postprocessed via phase correction. The instrument was shimmed every 15 min using IPA solvent. Peak referencing was performed on IPA’s methyl protons located in the upfield region at ∼1.2 ppm. Chemometric calibration was performed in JMP to obtain ^1^H NMR confidence intervals. A glass flow cell was installed for continuous online NMR analysis. The flow cell was oriented horizontally through the bore of the NMR and connected to the process with 1/16 in. tubing, with an internal analyzation volume of 0.583 cm^3^. The process stream was pumped through the base of the flow cell, through the analyzing region, and out from the top of the NMR using an Ismatec Reglo ICC peristaltic pump.

## Supplementary Material



## Notes

LC-MS analysis of amination products, representative HPLC chromatograms, UV–vis spectra of species, representative GC-FID chromatograms, ^1^H NMR spectra of key compounds, preliminary cost analysis, additional experimental details, materials, and methods, including photographs of the experimental setup (PDF)

## Abbreviations

AMT: Advanced Manufacturing Technology
API: Active Pharmaceutical Ingredient
CAS#: Chemical Abstract Services Number
cGMP: Current Good Manufacturing Practice
CI: Confidence Interval
CMA: Critical Material Attribute
CMO: Contract Manufacturing Organization
COPD: Chronic Obstructive Pulmonary Disorder
CPP: Critical Process Parameter
CQA: Critical Quality Attribute
C-SOPS: Center for Structured Organic Particulate Systems
CSTR: Continuously Stirred Tank Reactor
DAD: Diode Array Detector
DCS: Distributed Control System
DOE: Design of Experiments
DP: Drug Product
DS: Drug Substance
FDA: U.S. Food and Drug Administration
FID: Flame Ionization Detector
GC: Gas Chromatography
GLP: Good Laboratory Practices
HPLC: High-Performance Liquid Chromatography
HT: High-Throughput
ICH: International Council for Harmonization
IPA: Isopropyl Alcohol
LABA: Long-Acting Beta-Agonist
LFD: Laboratory Filter Dryer
LFR: Laminar Flow Reactor
LOD: Limit of Detection
MS: Mass Spectrometry
NMR: Nuclear Magnetic Resonance
NOR: Normal Operating Range
PAT: Process Analytical Technology
PFD: Process Flow Diagram
PFR: Plug Flow Reactor
RBF: Round Bottom Flask
RSM: Regulatory Starting Material
RTD: Resistance Temperature Detector
RTQC: Real-Time Quality Control
SABA: Short-Acting Beta-Agonist
SM: Starting Material
TFF: Tangential Flow Filter
UHP: Ultra High Purity
USP: United States Pharmacopeia
VLE: Vapor Liquid Equilibrium
wt.%: Weight Percent
L-WHSV: Liquid Weight Hourly Space Velocity

## References

[1] Sardella, A. US Generic Pharmaceutical Manufacturer Available Capacity Research Survey; Washington University, 2022.

[2] Socal M. P., Ahn K., Greene J. A., Anderson G. F. (2023). Competition
And Vulnerabilities In
The Global Supply Chain For US Generic Active Pharmaceutical Ingredients. Health Aff..

[3] Brainard, L. ; Sullivan, J. 2021–2024 Quadrennial Supply Chain Review; ADB, 2024.

[4] Sardella, A. The US Active Pharmaceutical Ingredient Infrastructure: the Current State And Considerations To Increase US Healthcare Security; 2021. https://www.whitehouse.gov/wp-content/uploads/2021/06/100-day-supply-chain-review-report.pdf.

[5] U.S. Food and Drug Administration. Report on the State of Pharmaceutical Quality FY2024; U.S. Food and Drug Administration, 2024.

[6] U.S. Food and Drug Administration. Current and Resolved Drug Shortages and Discontinuations Reported to FDA. https://www.accessdata.fda.gov/scripts/drugshortages/default.cfm. accessed 08-12-2025.

[7] U.S. Food and Drug Administration. Advanced Manufacturing Technologies Designation Program Guidance for Industry; 2024. https://www.fda.gov/regulatory-information/search-fda-guidance-documents/advanced-manufacturing-technologies-designation-program. accessed 19-05-2025.

[8] Hyer A., Gregory D., Kay K., Le Q., Turnage J., Gupton F., Ferri J. K. (2024). Continuous Manufacturing
of Active
Pharmaceutical Ingredients: Current Trends and Perspectives. Adv. Synth. Catal..

[9] Ciriminna R., Della Pina C., Luque R., Pagliaro M. (2025). The Fine Chemical Industry,
2000–2024. Org. Process Res. Dev..

[10] Pollak, P. Fine Chemicals The Industry and the Business; Wiley, 2011.

[11] Shah N. (2004). Pharmaceutical
Supply Chains: Key Issues and Strategies for Optimisation. Comput. Chem. Eng..

[12] Jaberidoost M., Nikfar S., Abdollahiasl A., Dinarvand R. (2013). Pharmaceutical
Supply Chain Risks: A Systematic Review. DARU
J. Pharm. Sci.

[13] Woodcock, J. Remarks by Acting Commissioner Janet Woodcock to the FDA Pharmaceutical Quality Symposium 2021: innovations in a Changing World; U. S. Food and Drug Administration, 2021.

[14] ICH Harmonised Tripartite Guideline. ICH Harmonised Tripartite Guideline Impurities in New Drug Substances Q3A(R2); ICH Harmonised Tripartite Guideline, 2006.

[15] Choe, J. ; Crane, M. ; Greene, J. ; Long, J. ; Mwanga, J. ; Sharfstein, J. M. ; Socal, M. ; Strodel, R. Pandemic And The Supply Chain; 2020. https://publichealth.jhu.edu/sites/default/files/2023-04/pandemic-supply-chain.pdf. accessed 18-02-2025.

[16] Ciriminna R., Della Pina C., Luque R., Pagliaro M. (2024). Reshoring Fine Chemical
and Pharmaceutical Productions. Org. Process
Res. Dev..

[17] Center For Structured Organic Particulate Systems (C-SOPS). https://www.c-sops.org. accessed 09-12-2025.

[18] U.S. Congress. Title 21- Food And Drugs; U.S. Congress: Washington D.C, 2016; pp 25–551. https://www.govinfo.gov/content/pkg/USCODE-2015-title21/pdf/USCODE-2015-title21-chap9-subchapV-partA-sec356e.pdf. accessed 19-02-2025.

[19] Colvill, S. ; Joyce, C. ; Roades, T. ; Hamre, G. Considerations for FDA’s New Advanced Pharmaceutical Manufacturing Programs; U.S. Food and Drug Administration, 2023. accessed 02-18-2025.

[20] Nelson, A. ; Koizumi, K. National Strategy for Advanced Manufacturing, 2022. http://www.whitehouse.gov/ostp.

[21] European Medicines Agency. ICH guideline Q13 Continuous Manufacturing of Drug Substances and Drug Products; European Medicines Agency, 2021.

[22] U.S. Food and Drug Administration. Guidance for Industry PAT - A Framework for Innovative Pharmaceutical Development, Manufacturing, and Quality Assurance; U.S. Food and Drug Administration, 2004. http://www.fda.gov/cvm/guidance/published.html.

[23] Nasr M. M., Krumme M., Matsuda Y., Trout B. L., Badman C., Mascia S., Cooney C. L., Jensen K. D., Florence A., Johnston C. (2017). Regulatory
Perspectives on Continuous Pharmaceutical
Manufacturing: Moving From Theory to Practice: September 26–27,
2016 International Symposium on the Continuous Manufacturing of Pharmaceuticals. J. Pharm. Sci..

[24] Romañach R. J., Stelzer T., Sanchez E., Muzzio F. (2023). Advanced Pharmaceutical
Manufacturing: A Functional Definition. J. Adv.
Manuf. Process..

[25] U.S. Food and Drug Administration. Drug Shortages; U.S. Food and Drug Administration, 2022. https://www.fda.gov/media/169302/download. accessed 10-02-2025.

[26] World Health Organisation. World Health Organization Model List of Essential Medicines; U.S. Food and Drug Administration, 2023. http://apps.who.int/bookorders.

[27] U.S. Food and Drug Administration. Drug Shortages Report, Root Causes and Potential Solutions; U.S. Food and Drug Administration, 2019.

[28] Vardanyan, R. ; Hruby, V. Synthesis of Essential Drugs. Elsevier, 2006, pp. 143–159. 10.1016/B978-0-444-52166-8.X5000-6

[29] Vardanyan, R. S. ; Hruby, V. J. Adrenergic (Sympathomimetic) Drugs. In Synthesis of Best-Seller Drugs; Elsevier, 2016, pp. 189–199. 10.1016/b978-0-12-411492-0.00011

[30] Howe R., Crowther A., Stephenson J., Rao B., Smith L. (1968). Beta-Adrenergic
Blocking Agents I. Pronethalol and Related N-Alkyl and N-Aralkyl Derivatives
of 2-Amino-1-(2-Naphthyl)­Ethanol. J. Med. Chem..

[31] Hartley D., Jack D., Lunts L., Ritchie A. (1968). New Class of Selective
Stimulants of Beta-Adrenergic Receptors. Nature.

[32] Brittain B., Farmer J., Jack D., Martin L., Simpson W. (1968). Alpha-[(t-Butylamino)­Methyl]-4-Hydroxy-m-Xylene-Alpha-1
Alpha3-Diol (AH.3365): A Selective Beta-Adrenergic Stimulant. Nature.

[33] Kitazawa Y., Langham M. (1968). Influence of an Adrenergic Potentiator on the Ocular
Response to Catecholamines in Primates and Man. Nature.

[34] Lands A., Arnold A., McAuliff J., Luduena F., Brown T. (1967). Differentiation
of Receptor Systems Activated by Sympathomimetic Amines. Nature.

[35] Bryan J. (2007). Ventolin Remains
a Breath of Fresh Air for Asthma Sufferers, After 40 Years. Pharm. J..

[36] Crompton G. (2006). A Brief History
of Inhaled Asthma Therapy Over the Last Fifty Years. Primary Care Respir. J..

[37] Marques L., Vale N. (2022). Salbutamol in the Management
of Asthma: A Review. Int. J. Mol. Sci..

[38] Bakale R. P., Wald S. A., Butler H. T., Gao Y., Hong Y., Nie X., Zepp C. M. (1996). Albuterol A Pharmaceutical
Chemistry Review of R-,
S-, and RS-Albuterol. Clin. Rev. Allergy Immunol..

[39] Hartley D., Middlemiss D. (1971). Absolute Configuration of the Optical Isomers of Salbutamol. J. Med. Chem..

[40] Hawkins C., Klease G. (1973). Relative Potency of
(−) and (+)-Salbutamol on
Guinea Pig Tracheal Tissue. J. Med. Chem..

[41] Ameredes B. T., Calhoun W. J. (2006). (R)-Albuterol Has
No Advantage over Racemic Albuterol. Am. J.
Respir. Crit. Care Med..

[42] Bartolinčić A., Drušković V., Šporec A., Vinković V. (2005). Development and Validation of HPLC Methods for the
Enantioselective Analysis of Bambuterol and Albuterol. J. Pharm. Biomed. Anal..

[43] Rogueda P., Lallement A., Traini D., Iliev I., Young P. M. (2012). Twenty
Years of HFA PMDI Patents: Facts and Perspectives. J. Pharm. Pharmacol..

[44] Sanders M. (2007). Inhalation
Therapy: An Historical Review. Primary Care
Respir. J..

[45] Clark A. R. (1995). Medical
Aerosol Inhalers: Past, Present, and Future. Aerosol Sci. Technol.

[46] Stein S. W., Thiel C. G. (2017). The History of Therapeutic
Aerosols: A Chronological
Review. J. Aerosol Med. Pulm. Drug Delivery.

[47] Anderson P. (2005). History of
Aerosol Therapy: Liquid Nebulization to MDIs to DPIs. Respir. Care.

[48] Gardenhire, D. S. ; Burnett, D. ; Strickland, S. ; Myers, T. R. A Guide To Aerosol Delivery Devices for Respiratory Therapists; American Association for Respiratory Care, 2017.

[49] Kazi A. A., Subba Reddy B. V., Ravithej Singh L. (2021). Synthetic Approaches to FDA Approved
Drugs for Asthma and COPD from 1969 to 2020. Bioorg. Med. Chem..

[50] Caves, R. E. ; Whinston, M. D. ; Hurwitz, M. A. Patent Expiration, Entry, and Competition in the U.S Pharmaceutical Industry; IICA, 1991.

[51] Schaber S. D., Gerogiorgis D. I., Ramachandran R., Evans J. M. B., Barton P. I., Trout B. L. (2011). Economic Analysis
of Integrated Continuous and Batch
Pharmaceutical Manufacturing: A Case Study. Ind. Eng. Chem. Res..

[52] Collin D., Hartley D., Jack D., Lunts L., Press J., Ritchie A., Toon P. (1970). Saligenin
Analogs of Sympathomimetic
Catechol amines. J. Med. Chem..

[53] Skachilova S. Y., Zueva E. F., Muravskaya I. D., Goncharenko L. V., Smirnov L. D. (1991). Methods for the Preparation of Salbutamol. Pharm. Chem. J..

[54] Lunts, L. H. C. ; Toon, P. Phenylaminoethanol derivatives; US 3,705,233 A, 1972.

[55] Lunts, L. H. C. ; Toon, P. 4 hydroxy-alpha’aminomethyl-m-xylene-alpha’ alpha-3-diols; US 3,644,353 A, 1972.

[56] Tann, C.-H. ; Thiruvengadam, T. K. ; Chiu, J. ; Green, M. ; Mcallister, T. L. ; Colon, C. ; Lee, J. Process for Preparing Albuterol, Acetal, Hemi-Acetal, and Hydrates of Arylglyoxal Intermediates Thereof; WO 1,992,004,314 A2, 1992.

[57] U.S. Food and Drug Administration. Approved Drug Products with Therapeutic Equivalence Evaluations; U.S. Food and Drug Administration, 2025.

[58] Babad E., Carruthers N. I., Jaret R. S., Steinman M. (1988). A Short Synthesis of
Albuterol. Synthesis.

[59] Oka, S. S. ; Escotet-Espinoza, M. S. ; Singh, R. ; Scicolone, J. V. ; Hausner, D. B. ; Ierapetritou, M. ; Muzzio, F. J. Design of an Integrated Continuous Manufacturing System. In Continuous Pharmaceutical Manufacturing; Wiley, 2017. DOI: 10.1002/9781119001348.ch12.

[60] Tao Y., Razavi S. M., Scicolone J. V., Ortega-Zúñiga C. A., Muzzio F. J. (2025). Effect of Tracer Material Properties and Tracer Amount
on RTD Characterization in Continuous Manufacturing. Powder Technol..

[61] Konkol J. A., Singh R., Muzzio F. J., Tsilomelekis G. (2024). On the Synthesis
of Diphenhydramine: Steady State Kinetics, Solvation Effects, and
in-Situ Raman and Benchtop NMR as PAT. Chem.
Eng. J..

[62] Kritikos A., Singh R., Tsilomelekis G., Muzzio F. J. (2024). A Novel CFD Model
of SMX Static Mixer Used in Advanced Continuous Manufacturing of Active
Pharmaceutical Ingredients (API). J. Pharm.
Innovation.

[63] Kritikos A., Singh R., Muzzio F., Tsilomelekis G. (2024). Inverse Method-Based
Kinetic Modelling and Process Optimization of Reverse-Phase Chromatography
for Molnupiravir Synthesis. Processes.

[64] Rezaeizadeh M., Razavi S. M., Muzzio F. J. (2026). Current
State of Machine Learning
Implementation in Pharmaceutical Process Modeling for Oral Solid Dosage
Forms. Int. J. Pharm..

[65] Carey, F. ; Sundberg, R. Functional Group Interconversion by Nucleophilic Substitution. In Advanced Organic Chemistry; Springer: Boston, MA, 1983, pp. 95–137. 10.1007/978-1-4757-1821-8_3

[66] Penzer, K. E. ; Gregory, D. G. ; Kohn, S. G. ; Kay, K. E. ; Turnage, J. T. ; Ferri, J. K. Advances in Continuous Manufacturing of Albuterol Sulfate: Optimization of an Amination Reaction in Flow. Reaction Chemistry and Engineering; **2026**.

[67] Turnage J., Gregory D. G., Ferri J. K. (2026). Tangential
Flow Filtration Enables
Continuous API Manufacturing: Case Study of Albuterol Sulfate. Chem. Eng. Sci..

[68] Brown, R. ; Stein, S. Boiling Point Data. In NIST Chemistry WebBook, NIST Standard Reference Database, Vol. 69, Linstrom, P. J. ; Mallard, W. G. , Eds.; National Institute of Standards and Technology: Gaithersburg MD, 2025

[69] Turnage, J. T. , Process Intensification of Separations Technologies for Continuous Pharmaceutical Manufacturing; Virginia Commonwealth University, 2024. M.S. Thesis 10.25772/ZAWT-CX45.

[70] Pearlman W. M. (1967). Noble Metal
Hydroxides on Carbon Nonpyrophoric Dry Catalysts. Tetrahedron Lett..

[71] Albers P. W., Möbus K., Wieland S. D., Parker S. F. (2015). The Fine
Structure
of Pearlman’s Catalyst. Phys. Chem. Chem.
Phys..

[72] Kay, K. E. ; Gregory, D. G. ; Gupton, B. F. ; Ferri, J. K. Continuous Hydrogenation in the Synthesis of Albuterol Sulfate. Adv. Synth. Catal. 2026.

[73] PharmaCompass. https://www.pharmacompass.com/active-pharmaceutical-ingredients/salbutamol-sulphate#allApiSuppliers. accessed 09-12-2025.

