# Supplementary material for: Development of an Advanced Manufacturing Technology for Continuous Drug Substance Production
Source: Ind Eng Chem Res. 2026 Jan 13;65(3):1469–82. doi: 10.1021/acs.iecr.5c04185 (PMC12856833; doi:10.1021/acs.iecr.5c04185)
Supplement: Supplementary file 1 [file ie5c04185_si_001.pdf]

Supplementary Information

Development of an Advanced Manufacturing Technology for Continuous Drug Substance Production

Daniel G. Gregory, Kaitlin E. Kay, Justin T. Turnage, Kimberly E. Penzer, James K. Ferri*

Department of Chemical and Life Science Engineering,
Virginia Commonwealth University, 601 West Main St., Richmond, VA 23284

SI Figures:

- **Figure S1.** Scheme depicting the potential species produced during Reaction Step #1: S_N2 Amination.
- **Figure S2.** LC-MS spectra corresponding to Table S1 of an amination product to demonstrate impurities.
- **Figure S3.** Potential amination impurities as detected with LC-MS.
- **Figure S4.** A representative HPLC chromatogram from the amination reaction.
- **Figure S5.** UV-Vis Spectra for (A) t-butylamine hydrochloride salt (CAS# 10017-37-5), (B) Intermediate (CAS# 15647-62-5), (C) albuterol sulfate (CAS# 51022-70-9).
- **Figure S6.** GC-FID Calibration Curves for tert-butylamine and isopropyl alcohol in acetonitrile.
- **Figure S7.** Representative HPLC chromatograms from the catalytic hydrogenation reaction when conducted in IPA at 60 °C, 10 Bar, and Q = 1.2 mL/min ($\tau \sim 1$ min) with the ThalesNano Reactor. The catalyst consisted of 20 wt% $Pd(OH)_2$/Carbon (0.25g). (Top) HPLC of the starting material prior to PBR entry. (Bottom) Product solution after catalytic reaction.
- **Figure S8.** LC-MS spectra corresponding to Table S2 of a hydrogenation product to demonstrate impurities.
- **Figure S9.** Simulated ^{1}H NMR shifts predicted by ChemDraw for key molecules including (I) the diol starting material, (II) the key intermediate, (III) albuterol freebase, (IV) isopropyl alcohol solvent, and the most abundant residual impurities: (V) t-butylamine, and (VI) dimerized byproducts.
- **Figure S10.** Key geminal and vicinal protons utilized for ^{1}H NMR analysis.
- **Figure S11.** ^{1}H NMR chemometric calibration curves with 95% confidence intervals for (A) diol starting material, (B) intermediate, (C) albuterol freebase, (D) t-butylamine.
- **Figure S12.** Multiplexing of an on-line 80 MHz Magritek Spinsolve NMR spectrometer within a pilot plant.
- **Figure S13.** Approximate material costs and potential upstream potential processing routes. Approximate prices were compiled in 2025 from Descartes Datamyne, online vendors including Ambeed (SM) and PharmaCompass (API).
- **Figure S14.** Catalytic hydrogenation scale up for the production of albuterol freebase over a granularized Pearlman's Catalyst with D_{50} = 150-190 um. Representative engineering test run for catalytic hydrogenation at 3.0 mL/min for +5 hours.
- **Figure S15.** A prototype distillation system and integration of the scale during the research and development.

SI Tables:

- **Table S1.** LC-MS analysis of an amination product (reaction step 1) which was collected during an early screening study (i.e., outside of optimal conditions) when first assessing the reaction impurities.
- **Table S2.** Comparison of continuous distillation experiments to binary distillation as simulated with ASPEN+ including the feed (F), distillate (D), and bottoms (B) composition in mole percent at associated temperatures. The simulation modeled a six-stage column with a distillate rate of 1.27 mol/hr and a reflux ratio of 1.2 to achieve the desired purity of <0.01 mole fraction of t-butylamine in the bottoms.
- **Table S3.** LC-MS analysis of a hydrogenation product sample (reaction step 2) as collected during an early screening study (i.e., outside of optimal conditions) when first assessing the reaction impurities. This data demonstrates the formation of albuterol and the potential hydrogenation of dimerized species.
- **Table S4.** Solubility limits of the Bromo-Diol Starting Material in key solvent mediums.
- **Table S5.** Solubility limits of albuterol sulfate in key solvent mediums.
- **Table S6.** Projected albuterol sulfate production rate for the prototype AMT a system with a basis of 1.0 mL/min.

Supplementary Information Discussion

During the first reaction step in the synthetic pathway as detailed within in this text (*i.e.*, S_N2 Amination), a bromo-diol starting material (CAS# 62932-94-9) is mixed with four equivalents of t-butylamine (CAS# 75-64-9) to form a target intermediate API species (CAS# 156547-62-5). This reaction is readily achieved at moderate temperatures, within dry solvents, and under inert N_2 atmosphere. However, when in the presence of air and moisture, this transformation is susceptible to oxidative degradation which results in a series of decomposed species (See **Figure S1**). Under prolonged reaction and increased temperatures, the reaction can continue while forming a series of tertiary amine dimers, trimers, and oligomers (See **Table S1** and **Figures S2-S3**). Oxidative decomposition can be prevented by operating under an inert blanket of nitrogen and ensuring that all glassware and solvents are free of residual moisture while process optimization was able to limit the amount of oligomerization that occurred (See **Figure S4**).

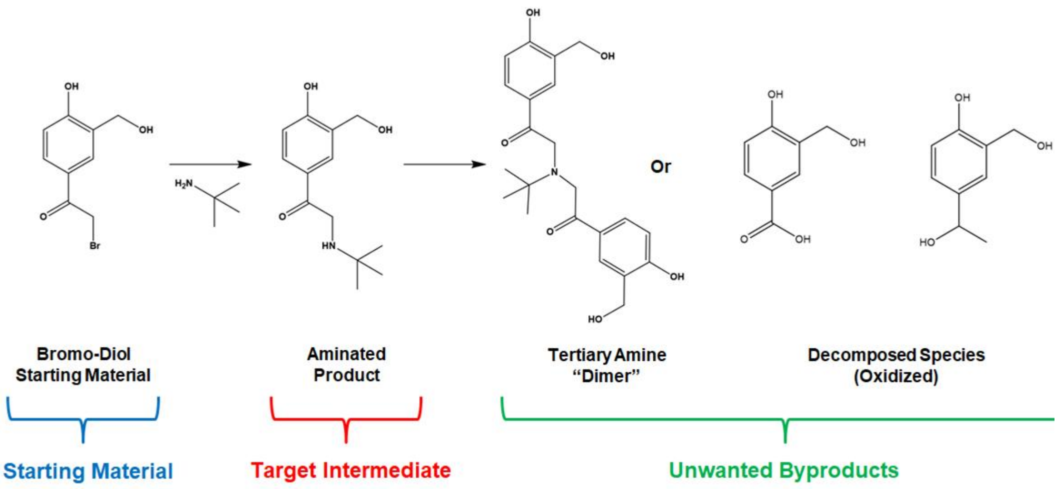

Figure S1. Scheme depicting the potential species produced during Reaction Step #1: S_N2 Amination.

Table S1. LC-MS analysis of an amination product (reaction step 1) which was collected during an early screening study (i.e., outside of optimal conditions) when first assessing the reaction impurities.

RT (min)	260 nm Area %	m/z	Comments
0.702	Not Detected	227 (ESI+); 232 (ESI-)	1 Br; 2 Br
2.670	76%	238 (M+H); 236 (M-H)	Key Intermediate
4.146	1.00%	457 (ESI+); 455 (ESI-)	Dimer
4.238	1.40%	457 (ESI+); 455 (ESI-)	Dimer
4.614	11.80%	402 (ESI+); 400 (ESI-)	Dimer
4.877	4.40%	402 (ESI+); 400 (ESI-)	Dimer
5.068	0.90%	199 (ESI-)	1 Cl
5.217	2.00%	238 (ESI+); 236 (ESI-)	Decomposed
5.480	0.80%	566 (ESI+); 564 (ESI-)	Trimer
5.947	0.20%	363 (ESI-)	1 Cl
6.929	0.09%	453 (ESI-)	1 Br
7.178	0.08%	617 (ESI-)	1 Br

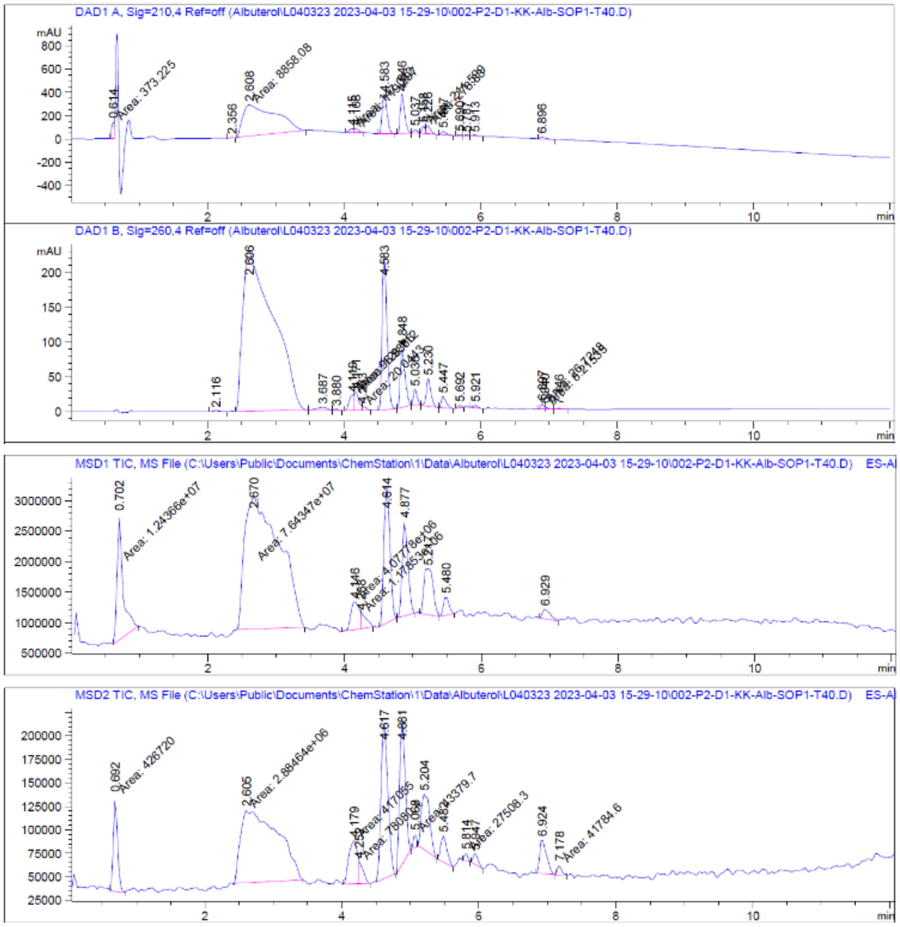

Figure S2. LC-MS spectra corresponding to Table S1 of an amination product which was collected during an early screening study outside of optimal conditions to demonstrate the common reaction generated impurities.

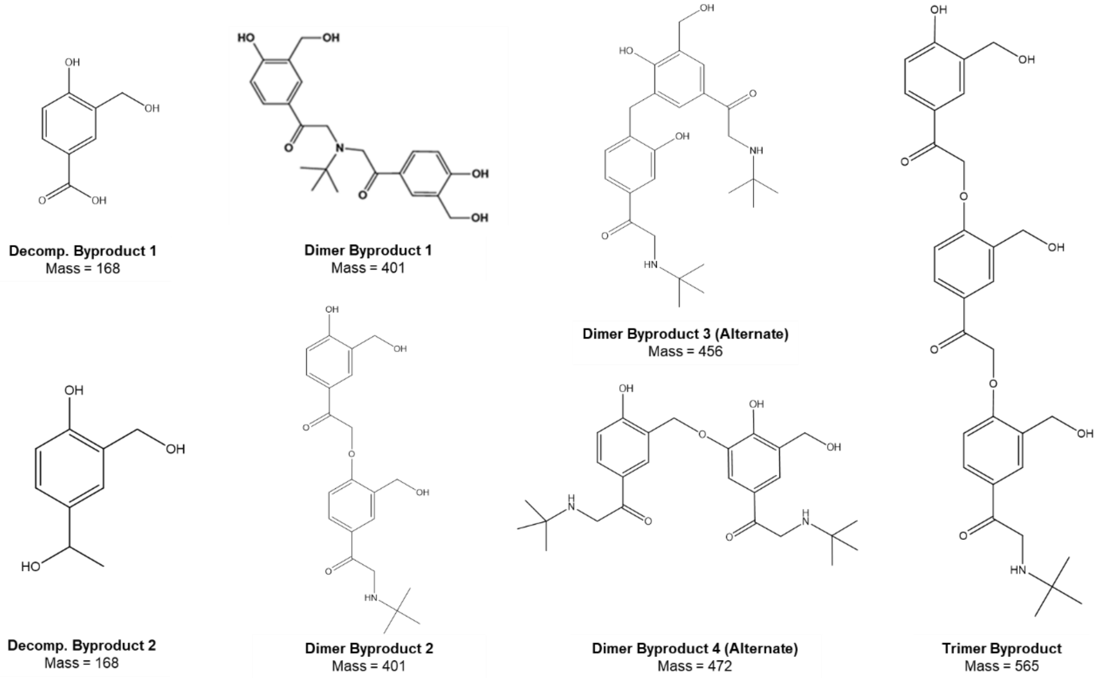

Figure S3. Amination impurities as detected with LCMS.

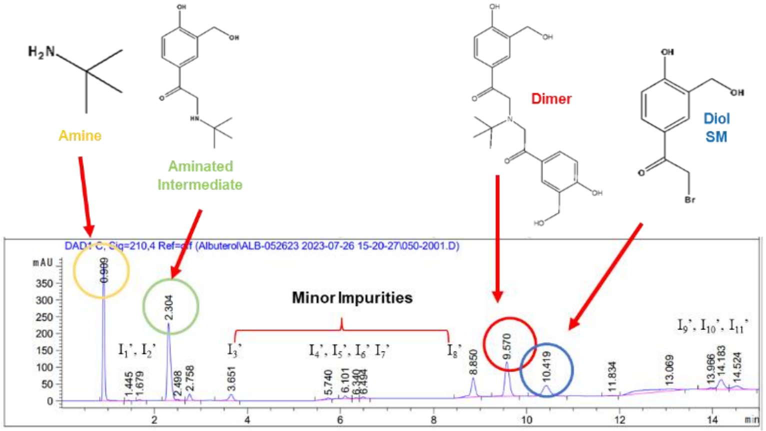

Figure S4. A representative HPLC chromatogram from the amination reaction when conducted in IPA at 60 °C (τ = 40 min) using the Vapourtec E Series reactor.

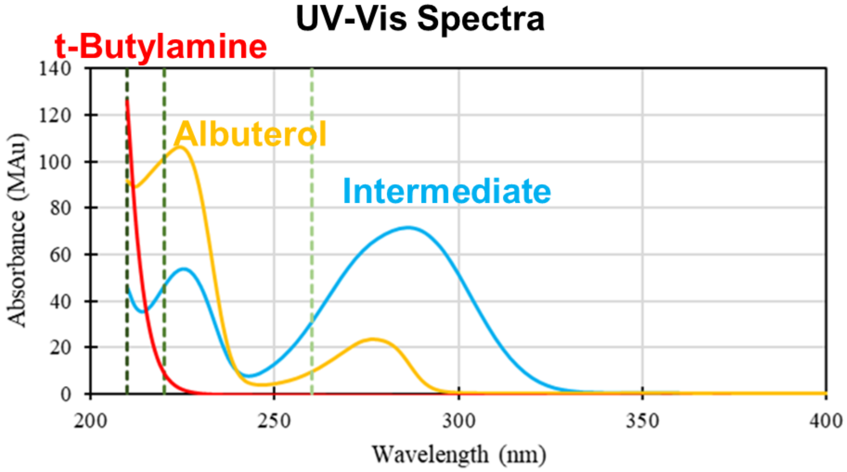

Figure S5. UV-Vis Spectra for (A) t-butylamine hydrochloride salt (CAS# 10017-37-5), (B) Intermediate (CAS# 15647-62-5), (C) albuterol sulfate (CAS# 51022-70-9).

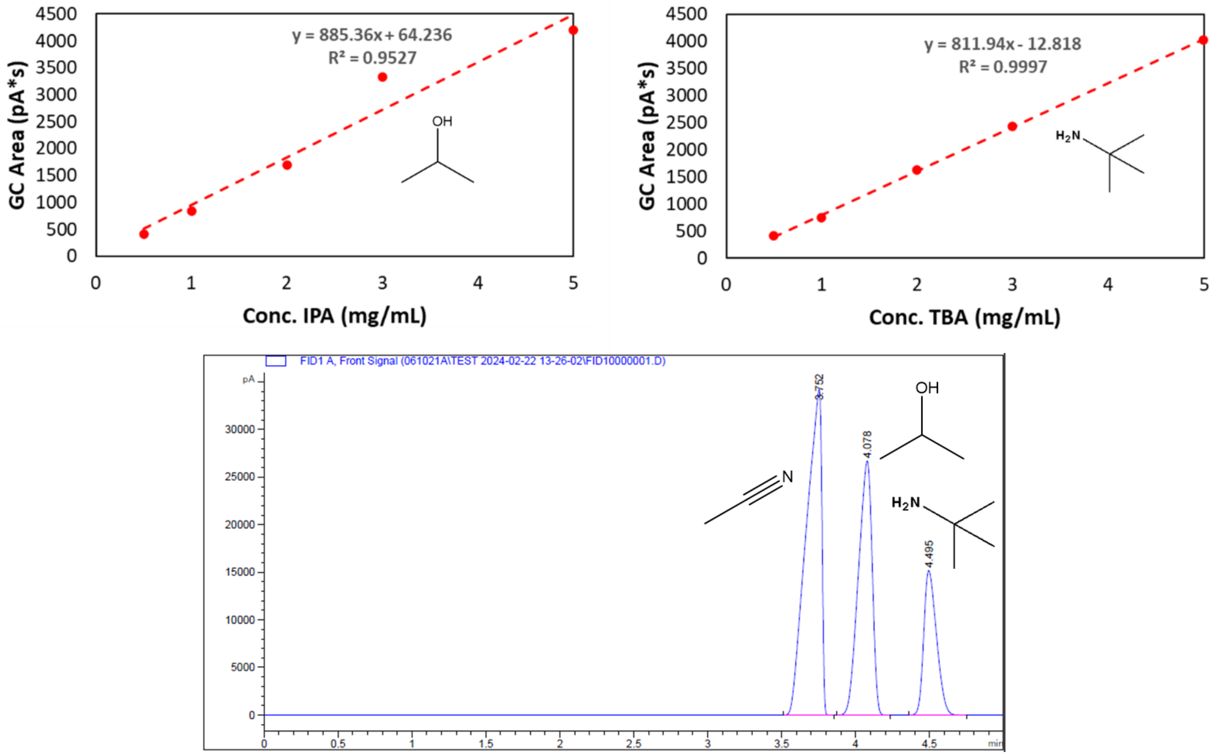

Figure S6. GC-FID Calibration Curves for tert-butylamine and isopropyl alcohol in acetonitrile (Top). Example chromatogram of species (Bottom).

Table S2. Comparison of continuous distillation experiments to binary distillation as simulated with ASPEN+ including the feed (F), distillate (D), and bottoms (B) composition in mole percent at associated temperatures. The simulation modeled a six-stage column with a distillate rate of 1.27 mol/hr and a reflux ratio of 1.2 to achieve the desired purity of <0.01 mole fraction of t-butylamine in the bottoms.

**Stream**	**TBA Composition (Mole%)**	**IPA Composition (Mole%)**	**Temperature (°C)**
Feed	8.0	92.0	-
Distillate	20	80.0	-
Bottoms	0.45	99.55	92.0

**Stream (ASPEN+)**	**TBA Composition (Mole%)**	**IPA Composition (Mole%)**	**Temperature (°C)**
Feed	3.20	96.80	80.3
Distillate	4.36	95.63	79.0
Bottoms	0.77	99.23	81.6

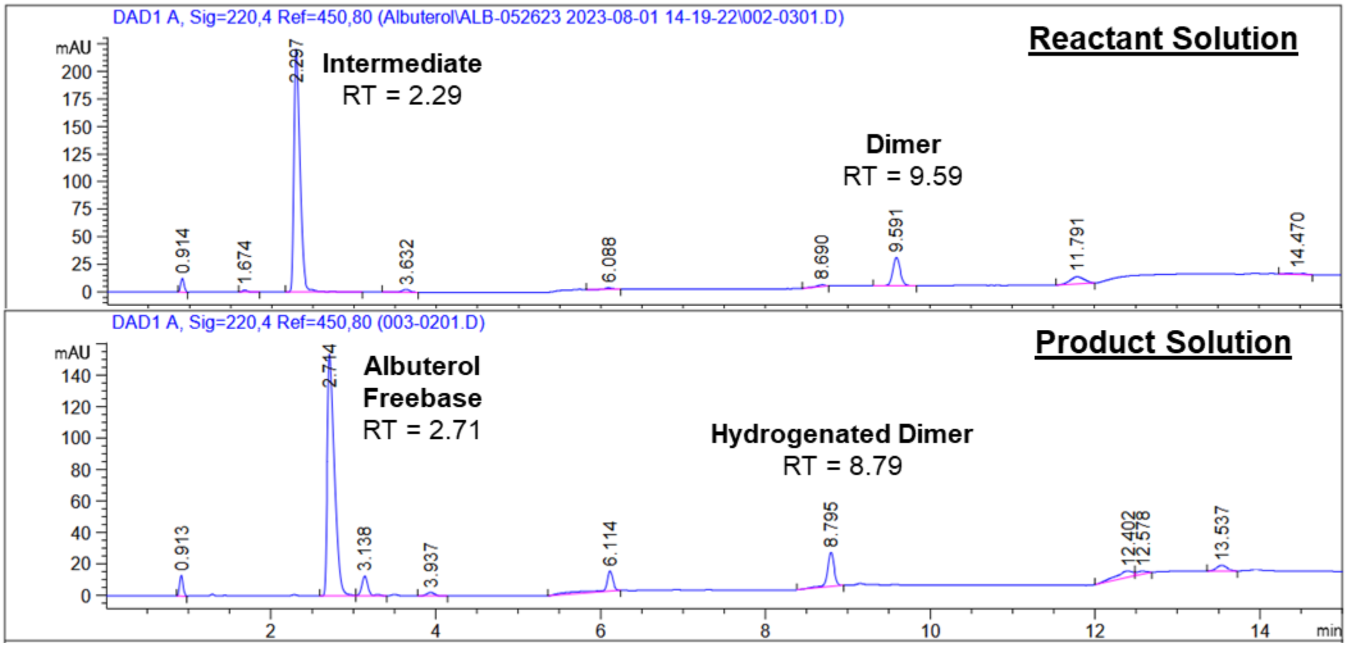

Figure S7. Representative HPLC chromatograms from the catalytic hydrogenation reaction when conducted in IPA at 60 °C, 10 Bar, and Q = 1.2 mL/min (τ ~ 1 min) with the ThalesNano Reactor. The catalyst consisted of 20 wt% $Pd(OH)_2$/Carbon (0.25g). (Top) HPLC of the starting material prior to PBR entry. (Bottom) Product solution after catalytic reaction.

Table S3. LC-MS analysis of a hydrogenation product sample (reaction step 2) as collected during an early screening study (i.e., outside of optimal conditions) when first assessing the reaction impurities. This data demonstrates the formation of albuterol and the hydrogenation of dimerized species.

LCMS Analysis: Hydrogenation Product Stream

RT (min)	260 nm Area %	m/z	Comments
0.711	Not Detected	227 (ESI+); 232 (ESI-)	1 Br; 2 Br
3.103	64%	240 (M+H); 238 (M-H)	Albuterol
3.646	4.00%	224 (ESI-)	
3.772	4.60%	167 (ESI-)	Decomposed Species
4.212	4.50%	224 (ESI+); 222 (ESI-)	Amination Dimer
4.351	10.80%	406 (ESI+); 404 (ESI-)	Hydrogenated Dimer
4.565	Not Detected	208 (ESI-)	
4.809	1.10%	390 (ESI+); 388 (ESI-)	
4.986	2.90%	390 (ESI+); 388 (ESI-)	

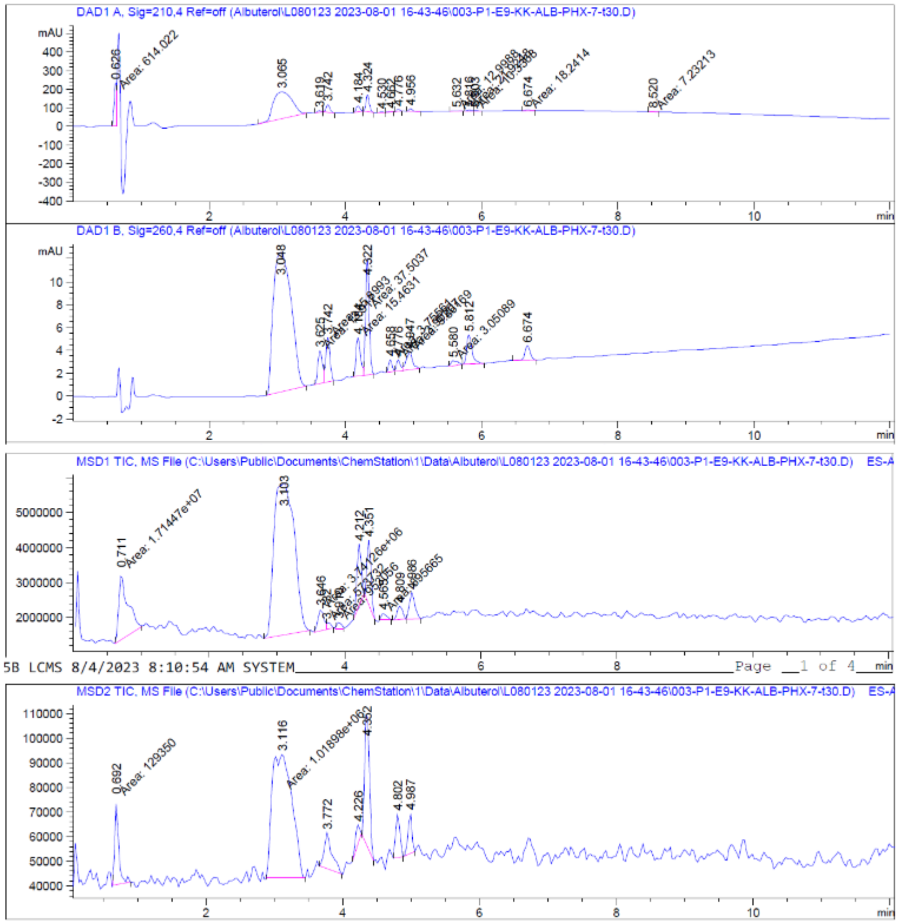

Figure S8. LC-MS spectra corresponding to Table S2 of a hydrogenation product which was collected during an early screening study outside of optimal conditions to demonstrate the common reaction generated impurities.

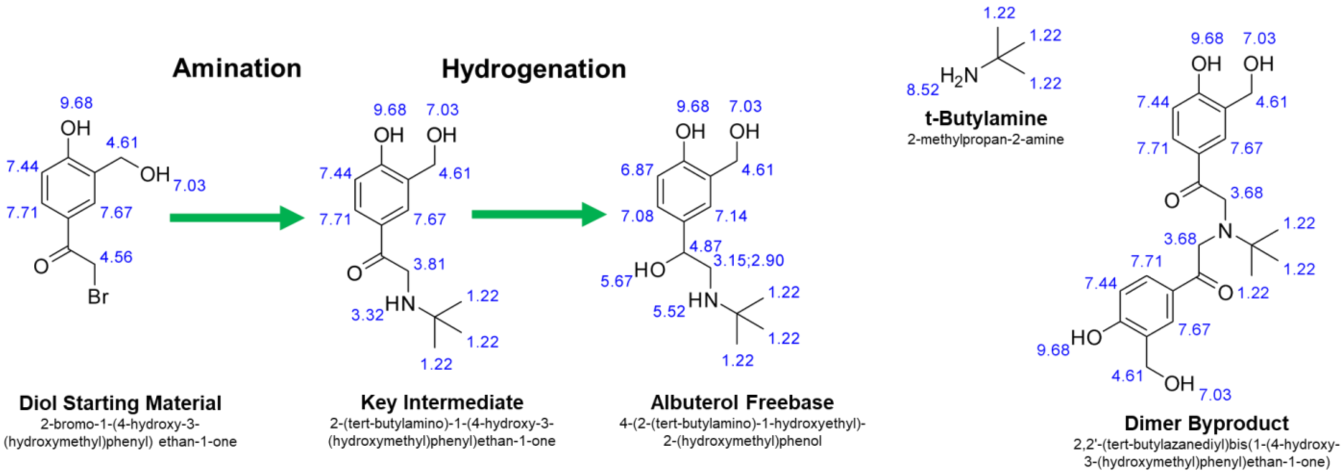

Figure S9. Simulated ^{1}H NMR shifts predicted by ChemDraw for key molecules including (I) the diol starting material, (II) the key intermediate, (III) albuterol freebase, (IV) isopropyl alcohol solvent, and the most abundant residual impurities: (V) t-butylamine, and (VI) dimerized byproducts.

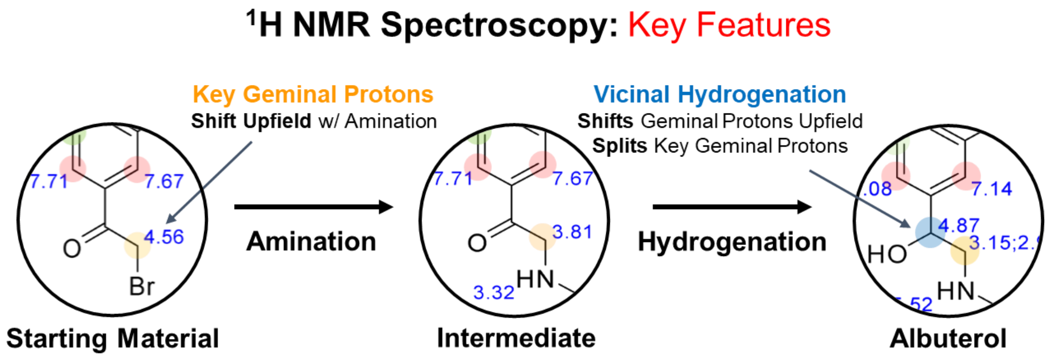

Figure S10. Key geminal and vicinal protons utilized for ^{1}H NMR analysis. The reported chemical shift was predicted by ChemDraw.

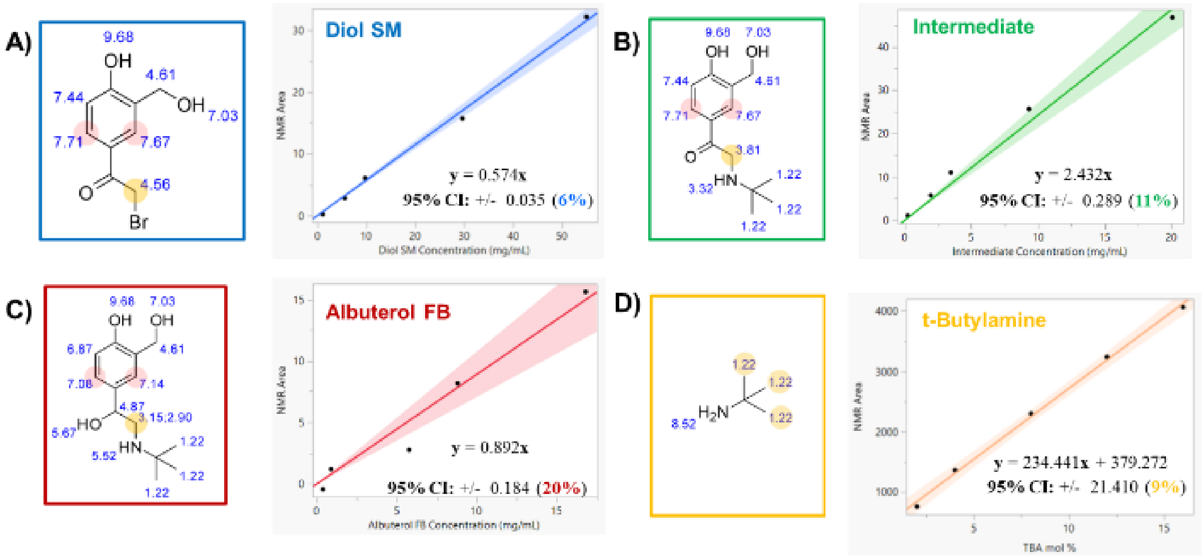

Figure S11. ^{1}H NMR chemometric calibration curves with 95% confidence intervals for (A) diol starting material, (B) intermediate, (C) albuterol freebase, (D) t-butylamine.

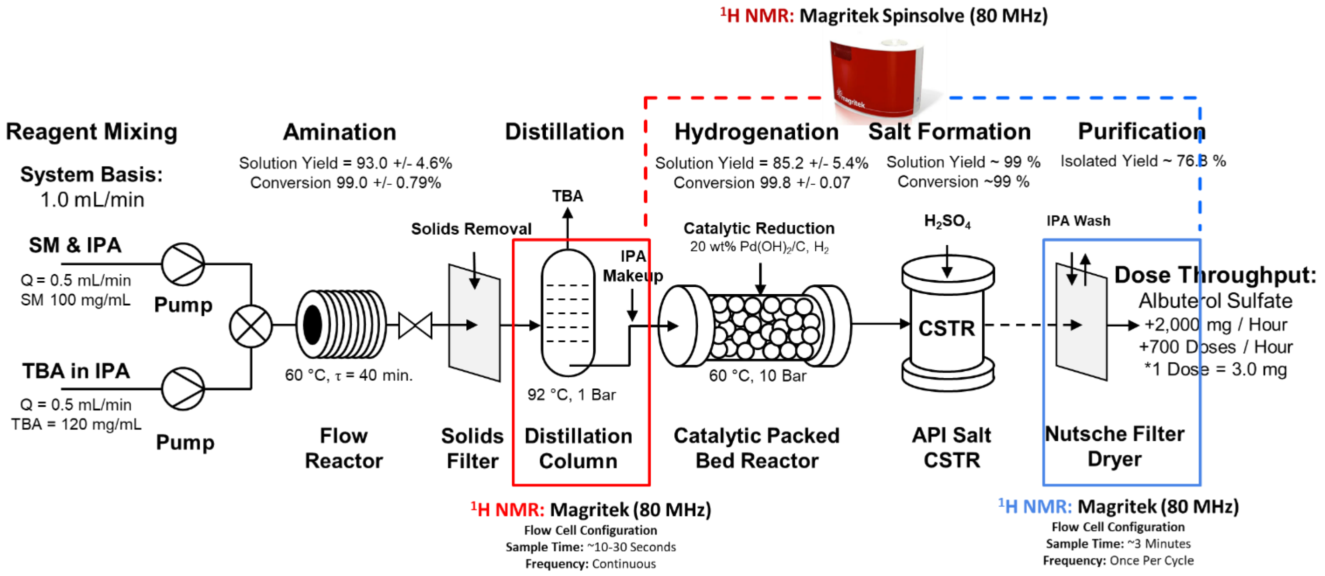

Figure S12. Multiplexing of an on-line 80 MHz Magritek Spinsolve NMR spectrometer within a pilot plant.

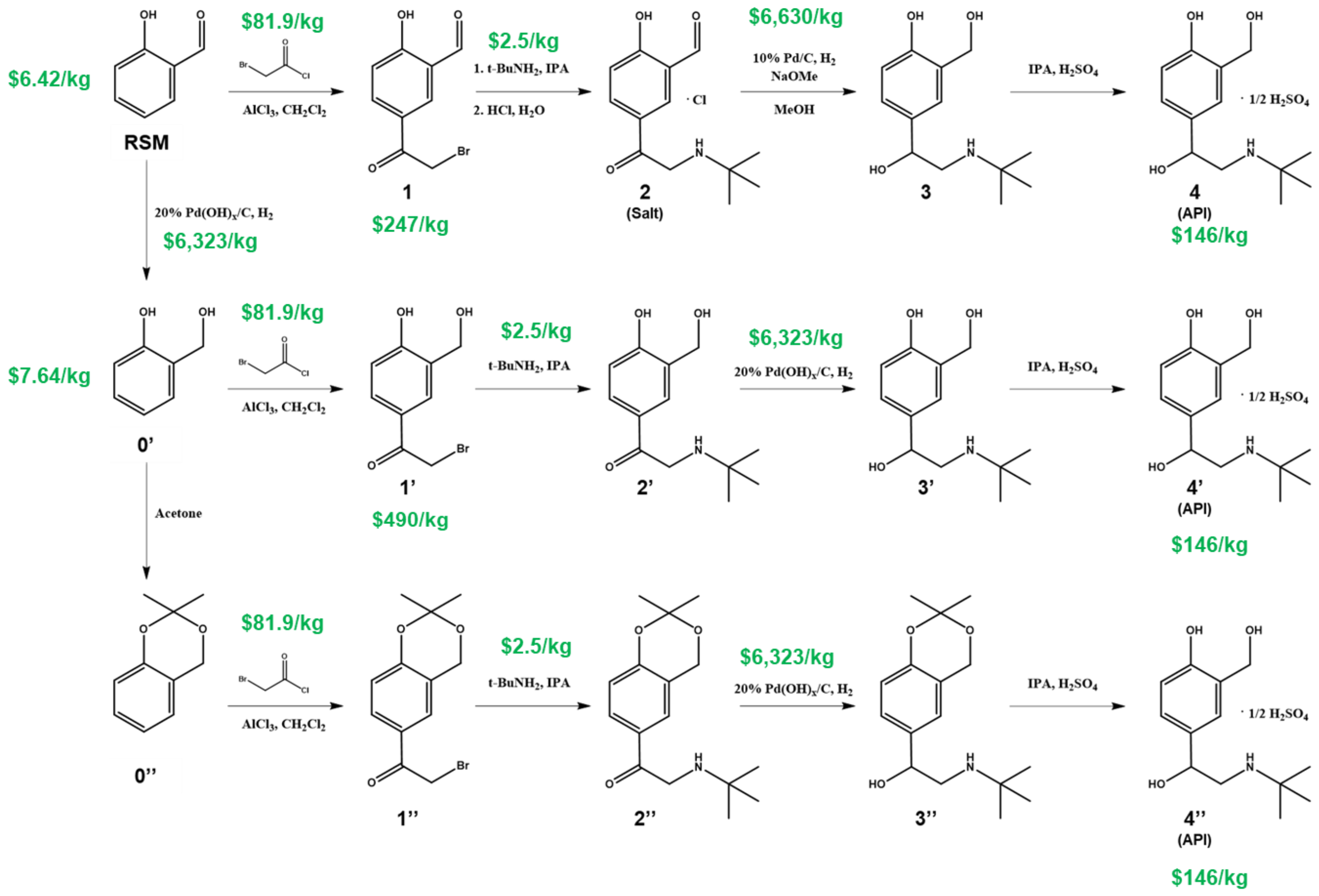

Figure S13. Approximate material costs and potential upstream potential processing routes. Approximate prices were compiled in 2025 from Descartes Datamyne, online vendors including Ambeed (SM) and PharmaCompass (API).
Web Source: https://www.pharmacompass.com

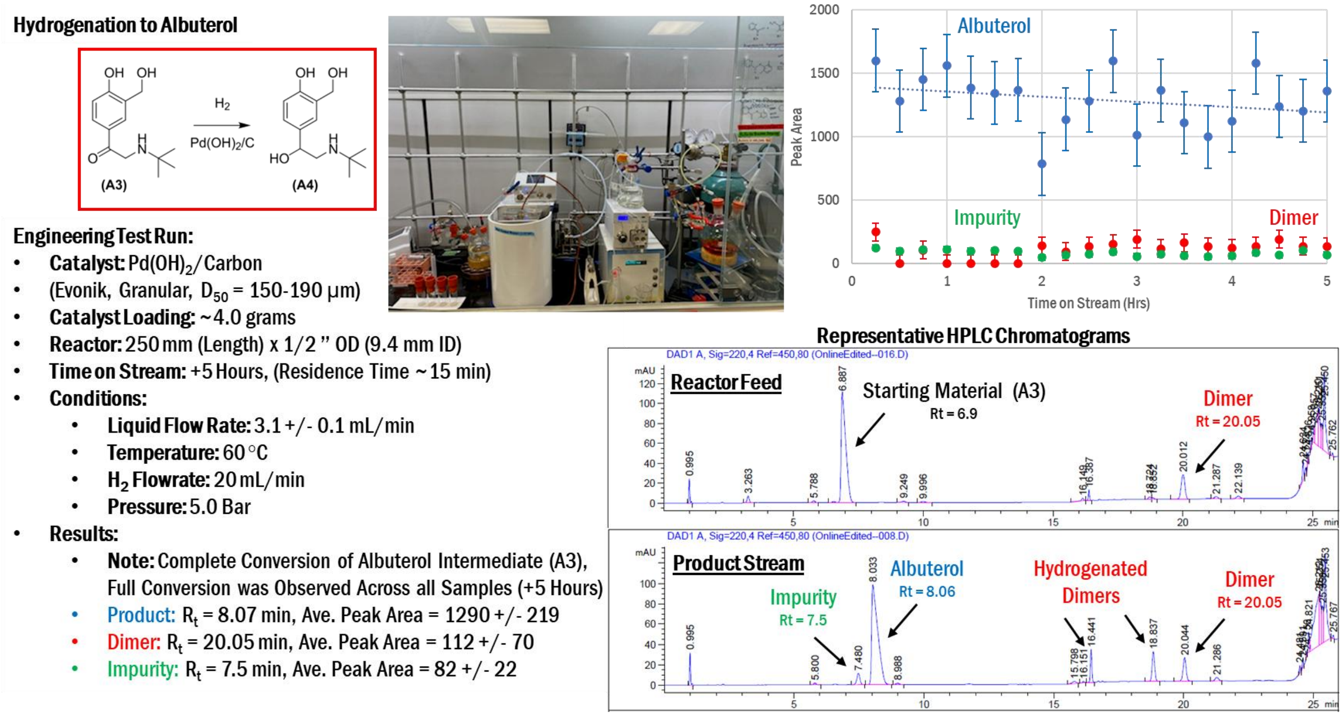

Figure S14. Catalytic hydrogenation scale up for the production of albuterol freebase over a granularized Pearlman's Catalyst with D_{50} = 150-190 um. Representative engineering test run for catalytic hydrogenation at 3.0 mL/min for +5 hours.

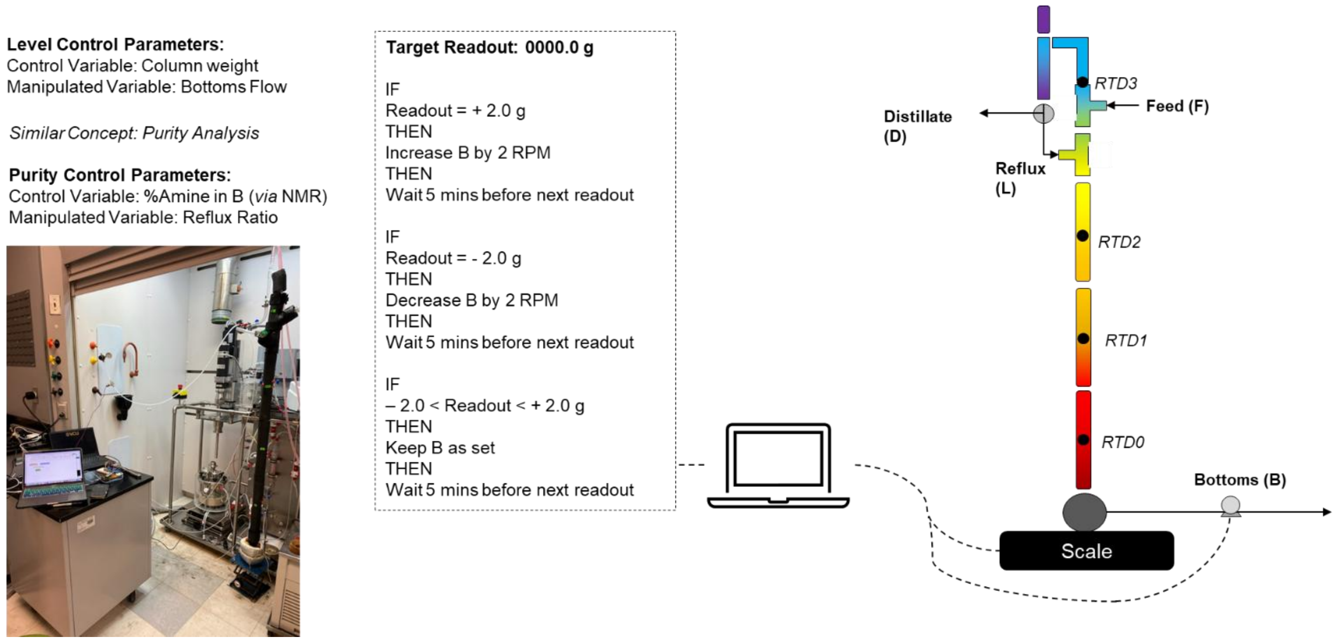

Figure S15. A prototype distillation system and integration of the scale during the research and development.

Table S4. Solubility limits of the Bromo-Diol Starting Material in key solvent mediums.

Solute	Solvent	Temp.	Concentration	Weight % Solute
Bromo-Diol SM	Methanol	RT	120 mg/mL	13.2 wt%
Bromo-Diol SM	Methanol	40°C	190 mg/mL	19.4 wt%
Bromo-Diol SM	IPA	RT	50 mg/mL	5.9 wt%
Bromo-Diol SM	IPA	40°C	120 mg/mL	13.2 wt%

Table S5. Solubility limits of albuterol sulfate in key solvent mediums.

Solute	Solvent	Weight % Solute
Albuterol Sulfate	Deionized Water	16.7 ± 1.0
Albuterol Sulfate	Methanol	0.85 ± 0.01
Albuterol Sulfate	Ethanol	0.175 ± 0.025
Albuterol Sulfate	Isopropyl Alcohol	0.0225 ± 0.0025

Table S6. Projected albuterol sulfate production rate for the prototype AMT a system with a basis of 1.0 mL/min. A month is defined here as 30 days. A half month is defined as 15 days. This throughput assumes no down time.

**System Basis:** 1.0 mL/min	**API Sulfation**	**API Purification**
**Mass Flow Rate** (g/Hr)	2.77	2.13
**Mass Flow Rate** (g/Day)	64.5	51.0
**Mass Flow Rate** (kg/Half Month)	1.00	0.77
**Mass Flow Rate** (kg/Month)	1.99	1.53

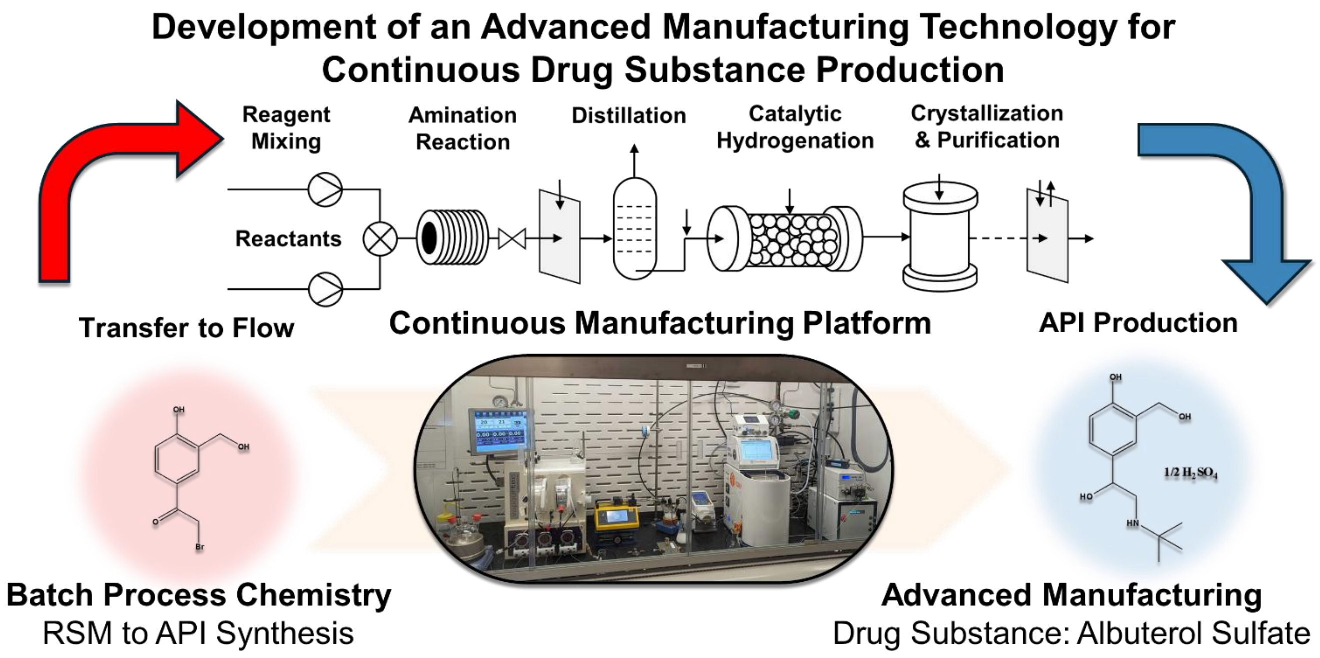

TOC Figure Development of an Advanced Manufacturing Technology for Continuous Drug Substance Production (*i.e.*, Albuterol Sulfate).
